# Structural Bases for the Unconventional Activity of a Viroporin Channel

**DOI:** 10.1021/acs.biochem.6c00159

**Published:** 2026-06-25

**Authors:** Brian Wiley, Eneko Largo, Laura Nabais, José L. Nieva, Carmen Domene

**Affiliations:** † Department of Chemistry, 1555 University of Bath, Claverton Down, Bath BA2 7AX, U.K.; ‡ Department of Immunology, Microbiology and Parasitology, Medicine and Odontology Faculty, University of Basque Country (EHU), PO Box 644, Bilbao 48080, Spain; § Instituto Biofisika (CSIC, EHU), University of the Basque Country (EHU), P.O. Box 644, Bilbao 48080, Spain; ∥ Department of Biochemistry and Molecular Biology, University of the Basque Country (EHU), P.O. Box 644, Bilbao 48080, Spain

## Abstract

Viroporins alter the permeability of cell membranes and regulate the initiation/progression of the viral infection cycle. However, the “unconventional” membrane channel behavior displayed by many members of the family challenges their general validation as therapeutic targets. The reported capacity of the Classical Swine Fever Virus p7 viroporin for establishing ion-conducting channels of different sizes exemplifies that behavior. Using all-atom molecular dynamics (MD) simulations, we attempted to elucidate the structural basis and mechanisms underlying the atypical activity of p7. Based on AlphaFold-predicted CSFV p7 hexamer structures with folded-back helical hairpins, we first generated monomers that spanned the entire thickness of the lipid bilayer. Next, we assembled oligomers of varying stoichiometry (pentamers, hexamers, and heptamers) based on those extended transmembrane hairpin (TMH) protomers. We focused on two hexameric models: TMH1, preserving the helix–helix packing interactions observed in the initial model, and TMH2_6_, generated using TMH1 as a template for ColabFold. In line with experimental evidence, the simulations revealed that both structural architecture and oligomeric state determine pore organization in ER-like membranes. TMH1 hexamers and TMH2_7_ heptamers adopted wide pore geometries with extensive hydration and ion accessibility, whereas TMH2_6_ hexamers sampled narrower, more compact hydrated pore states. TMH2_5_ pentamers remained predominantly nonconductive. Dynamic interactions between transmembrane helices and the number of protomers incorporated into the membrane-embedded structure appeared to be instrumental for this capacity. These findings reveal how variation in oligomeric state and helix packing can generate pores with different dimensions and hydration properties, providing a structural framework for interpreting p7′s experimentally observed capacity to induce multiple conductance states and size-selective membrane permeabilization.

## Graphical abstract

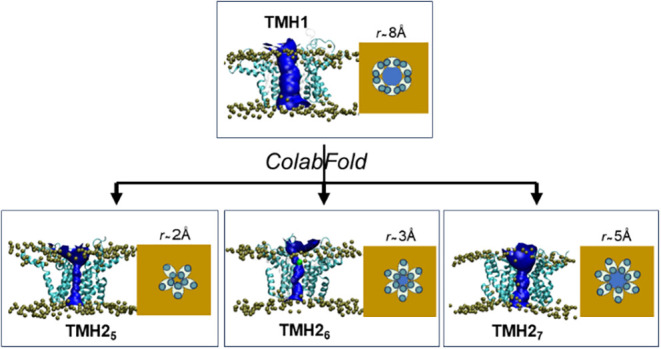

## Introduction

Small size (usually <10 kDa), high degree of hydrophobicity, and capacity for assembling oligomeric pores in membranes are defining features of prototypic viroporins.^1–5^ This family of integral membrane proteins comprises relevant therapy targets that play regulatory functions during virus infection, subverting cell biosynthetic pathways and promoting new viral progeny production.^6–9^ However, structure–function relationships remain undefined for many members of the viroporin family, precluding the formulation of a unified mechanism that explains the implications of viroporin-induced membrane permeabilization for cell function.^3^ In that regard, the “unconventional” behavior as membrane ion channels,^10^ a functional trend described for a broad spectrum of family members,^3,11–15^ remains a controversial issue that hinders the development of therapies based on these viral proteins.

The function-related features of the “unconventional” ion channels include a large heterogeneity in the conductive levels, poor ion selectivity and dependence on lipid composition, properties that usually combine with the ability of the protein to permeabilize lipid vesicles to small solutes.^3,10,11^ It has been argued that the capacity to form a variety of flexible membrane structures, in which even lipids may be involved, could be at the origin of this unconventional ion-channel activity.^3,15^ Thus, even though available structures adopted by notorious members of this family such as Influenza A virus (IAV) M2 ^16,17^ or Coronavirus (CoV) E,^18–20^ support the assembly of defined membrane channel architectures, occurrence of these structures does not appear to be the rule; many viroporin-induced conducting pores may exhibit backbone conformational flexibility and constraints induced through direct interactions with the surrounding membrane (reviewed in ref 3).

Accumulated experimental work has typified the activity of the classical swine fever virus (CSFV) p7 viroporin as representative of unconventional ion channels.^13,21,22^ Supporting this categorization, CSFV p7 formed ion channels in planar lipid membranes that exhibited alternative current states, whose relative contribution to overall ion conduction was modulated by the lipid composition.^13,21^ Furthermore, those channels displayed mild selectivity and no ion specificity, and, consistently, CSFV p7 also induced dye leakage from unilamellar lipid vesicles of similar compositions.^21,22^ However, the lack of structural information about this viroporin has made it difficult to understand the molecular mechanism underlying these unconventional ion-channel and pore formation activities.

AlphaFold has proven useful for predicting 3D models,^23,24^ including those of integral membrane proteins, which are otherwise difficult to isolate and determine experimentally at atomic resolution. Appraisal through MD simulation methods and alignment with the available biochemical and biophysical evidence support the relevance of viroporin models predicted using this tool.^15,25–27^ In a recent report,^25^ Cheng and Wang describe AlphaFold-predicted transmembrane hairpin conformations in CSFV p7-family proteins, including hexameric assemblies, comprising protomers predicted to adopt folded-back helical hairpins that are not expected to span the lipid bilayer. Similar double-hairpin transmembrane predictions have also been reported across related hepacivirus and pestivirus viroporins, suggesting that this fold may represent a recurring structural tendency rather than a prediction artifact.^28^ Furthermore, these structures differed in important ways from the α-helix–turn−α-helix transmembrane hairpin (TMH) CSFV p7 model initially proposed by Gladue et al.,^29^ which was based on experimental evidence and sequence-based structure prediction tools available at the time.

Although hexameric assemblies have frequently been considered for CSFV p7, largely by analogy with HCV p7 and/or based on WB analyses and on previous computational studies, no experimentally determined oligomeric state has been established for CSFV p7, and the biologically relevant stoichiometry therefore remains unresolved. Accordingly, we examined pentameric, hexameric, and heptameric assemblies in the present work. Since CSFV p7 is inserted, folded, and oligomerized in the endoplasmic reticulum (ER) during the viral life cycle prior to further trafficking, experimental and computational analyses were performed in ER-like membrane environments. This choice was intended to reflect both the expected site of early viroporin assembly and the lipid composition relevant to p7 oligomerization and pore formation.

Here, we used a short-helix bundle AlphaFold model to produce extended TMHs capable of spanning the lipid bilayer and to infer helix–helix interactions that may underpin stable oligomeric p7 pore structures in membranes. On the basis of these monomers and helix packing interactions, we generated pentamers, hexamers, and heptamers whose pore formation dynamics during MD simulations are compatible with the unconventional ion and solute conductance experimentally observed for CSFV p7. Elucidating the structural basis and dynamics of this phenomenon may provide insights into new pathways for blocking viroporins in infectious disease-related settings.

### Materials and Methods

The CSFV p7 protein (Brescia isolate) was produced by solid-phase synthesis using Fmoc chemistry as C-terminal carboxamide and purified by HPLC. 1-Palmitoyl-2-oleoyl-glycero-3-phosphocholine (POPC), 1-palmitoyl-2-oleoyl-glycero-3-phosphoethanolamine (POPE), and 1-palmitoyl-2-oleoyl-glycero-3-phosphoinositol (POPI) were purchased from Avanti Polar Lipids (Birmingham, AL, USA). Alexa Fluor 488 and N-(lissamine rhodamine B sulfonyl) phosphatidylethanolamine (-Rho-PE) fluorescent probes were purchased from Thermo Fisher Scientific (Waltham, MA, USA). FITC-labeled fluorescent dextrans were purchased from Sigma-Aldrich (St. Louis, MO, USA).

### Membrane Permeability of Single Vesicles

Single-vesicle permeability measurements were performed on POPC:POPE:POPI:Rho-PE (50:30:20:0.1 mol ratio) Giant Unilamellar Vesicles (GUVs) prepared according to the electroformation method as described in previous works.^30,31^ In brief, 2 μL of the lipid mixture (2 mM) was placed on platinum wires and the solvent evaporated. For GUV electroformation, 2.4 V, 10 Hz was applied during 2 h in a sucrose (300 mM) solution. To promote detachment of the GUVs from the wires, 2 Hz was finally applied for 30 min. For confocal microscopy analyses, 80 μL of the solution was transferred to 320 μL of buffer (HEPES 10 mM, KCl 150 mM, pH 7.4) in a Lab-Tek eight-chambered #1.0 borosilicate cover glass from Nalge Nunc International (Rochester, NY, USA) previously blocked with 2 mg/mL BSA.

Subsequently, 0.15 mM Alexa Fluor 488 or different-size FITC-Dextrans and p7 (0.2 μM) were added to GUVs, gently mixed, and incubated for 30 min before imaging. Confocal images were acquired using a Leica TCS SP5 microscope. Vesicle permeabilization percentage was calculated based on the intensity of Alexa Fluor 488 or FITC-Dextran inside the vesicles. Fluorescence emission in the different regions of the sample was quantified with ImageJ software (rsb.info.nih.gov/ij/).

### Molecular Modeling

#### Model Generation

To distinguish the different oligomeric arrangements analyzed in this study, we designated the manually extended hexamer generated from the AlphaFold scaffold as TMH1. Subsequent ColabFold predictions obtained using this extended scaffold as a template yielded alternative oligomeric organizations, including the high-confidence TMH2_5_ pentamer, the TMH2_6_ hexamer, and the TMH2_7_ heptamer (Table 1).

**Table 1:** Overview of CSFV p7 Structural Models, Including a Literature-Based Model and AlphaFold-Derived Conformations Generated in This Study, Used to Probe the Effects of Topology, Orientation, and Prediction Confidence (pLDDT) on Pore Formation and Stability

model	notes	generation	description
TMH1	extended TMH hexamer	manually extended hexamer based on AlphaFold scaffold	hexamer generated by extending the AlphaFold-derived monomers while preserving h2–3 hairpin interactions; forms wide hydrated pores (6–8 Å)
TMH2_5_	high_pLDDT pentamer	ColabFold v1.6.1	extended monomers with high confidence regions; stable narrow pores
TMH2_6_	high_pLDDT hexamer	ColabFold v1.5.5	extended monomers with high confidence regions; stable narrow pores (∼3 Å)
TMH2_7_	high_pLDDT heptamer	ColabFold v1.6.1	high-confidence heptamer generated from extended monomers; forms hydrated pores with radii of ∼2–5 Å
TMH2_6_ **L**	**L**ow-confidence ColabFold hexamer control	ColabFold v1.5.5	TMH2-derived low-confidence variant; unstable and lacks persistent pore formation
TMH2_6_ **R**	**R**otated TMH2 control	ColabFold v1.5.5 + manual intervention	reverse-orientation control derived from TMH2; used to assess topology robustness and pore stability.
Gladue model	Gladue et al. hairpin	literature-derived^22^	hairpin-based model derived from previous studies; collapses during MD simulations and does not maintain stable pores

The folded transmembrane hairpin model of the p7 hexamer (Figure S1a) was generated using the first released version of AlphaFold.^23^ To construct alternative extended conformations, dihedral angles at the h1–h2 and h3–h4 connecting turns were modified to produce an extended monomer. Specifically, two targeted adjustments were introduced. In the first turn, the ψ dihedral angle of His17 was changed from 134.6 to −47.3°, a modification compatible with a transition of the main chain from an extended geometry toward a helical conformation. In the second turn, the ψ angle of Asn51 was altered from 21 to −172°, preserving a left-handed α-helical conformation while reorienting the Cα backbone direction. Importantly, these dihedral modifications did not introduce steric clashes between residues. The resulting monomers maintained their extended conformations after insertion into the endoplasmic reticulum lipid bilayer (ER-LB) during preliminary MD simulations (Figure S1b,d).

Six extended monomers were fitted into the h2–h3 hairpins of the hexamer to generate an extended TMH1 hexamer, which was subsequently used as a template for ColabFold^24^ operating in template mode. The residue sequence of the monomer was repeated six times to match the hexameric arrangement, and the resulting hexamer template was incorporated into multiple sequence alignments for HMMER,^32^ a tool that uses profile Hidden Markov Models to identify and align homologous sequences, as part of ColabFold’s template mode. ColabFold was executed using default parameters unless otherwise noted.

By default, ColabFold generates five structural models (num_models = 5) and applies a recycling procedure with a default recycle count of three (num_recycles = 3) to iteratively refine the predicted structures. The maximum number of recycling iterations for relaxation (relax_max_iterations) was set to 200, and early stopping was disabled (recycle_early_stop_tolerance = 0.0). Amber relaxation, which uses the AMBER molecular mechanics force field to relieve steric clashes and improve the local geometry of predicted structures, was not performed in our workflow (num_relax = 0) to retain the raw AlphaFold predictions for downstream MD simulations. Out of the five resulting models, two were selected for subsequent MD simulations: one prediction with a high pLDDT score (∼83.1; TMH2_6_ model) and another prediction with a substantially lower pLDDT score (52.4; Table 1, TMH2_6_L model), where pLDDT (predicted Local Distance Difference Test) is AlphaFold’s per-residue confidence metric, with higher values indicating greater confidence in the predicted local structure.

In addition, a rotated version of the high-pLDDT prediction was considered, generated by rotating each monomer by 180 deg (Table 1, TMH2_6_R model). All other parameters were kept at their default values: multiple sequence alignment generation mode (msa_mode = “mmseqs2_uniref_env”), model type (model_type = “alphafold2_multimer_v3”), number of random seeds (num_seeds = 1), dropout usage (use_dropout = False), model evaluation order (model_order = [1,2,3,4,5]), multimeric complex mode (is_complex = True), pair mode (pair_mode = “unpaired_paired”), pairing strategy (pairing_strategy = “greedy”), prediction stopping score (stop_at_score = 100.0), image resolution (dpi = 200), maximum MSA depth (max_msa = “auto”), cluster profile usage (use_cluster_profile = False), and calculation of extra PTM information (calc_extra_ptm = False).

For the generation of the TMH2_5_ pentamer model, we followed the same ColabFold modeling procedure, with the monomer sequence repeated five times and the TMH1 hexamer used as the template in template mode. For the generation of the TMH2_7_ heptamer model, the sequence of the extended monomer was repeated seven times and submitted to ColabFold using the same template-based workflow and parameters described above. The resulting high-confidence heptameric assembly was subsequently selected for the MD simulations.

Finally, a p7 hexamer was set to test the feasibility of pore formation by the hairpin model previously proposed by Gladue et al.^29^ (Table 1, Gladue model). This model was developed by integrating experimental evidence identifying the C-terminal helices h3 and h4 as the pore-forming domain with computational prediction tools, including TMHMM, Memsat, and SPOCTOPUS for membrane-spanning helices; SAM and PSI–PRED for secondary structure prediction; and Rosetta Membrane for three-dimensional modeling in a membrane environment.^22,29^ To evaluate this architecture, we generated hexamers based on this early model by starting from the extended hexamer and using MODELER to enforce straight α-helical conformations for residues Asn13–Met33 (h2) and Glu36–Gly66 (h3–h4) in each of the six monomers, consistent with the Gladue hairpin arrangement (Figure S3).

#### System Setup

To perform our MD studies in a function-supporting membrane system, we constructed an atomistic model of the ER membrane (Figure S1b). The small thickness and loose lipid packing of the ER membrane depend on the low packing density of the acyl chains in the organelle.^33^ Therefore, for each system, an ER-like membrane patch was constructed with X-Y dimensions matching the corresponding simulation box dimensions reported in Table 2. The ER-like lipid bilayer (ER-LB) system was generated using the Membrane Builder module of CHARMM-GUI.^34^ Each membrane consisted of 1-palmitoyl-2-oleoyl-*sn*-glycero-3-phosphocholine (POPC), 1-palmitoyl-2-oleoyl-*sn*-glycero-3-phosphoethanolamine (POPE), and 1-palmitoyl-2-oleoyl-*sn*-glycero-3-phospho-(1′-myo-inositol) (POPI) in a 5:3:2 molar ratio.^35^ As shown in Figure S1b, the ER-LB thickness (phosphate-to-phosphate mean distance of ∼39 Å), closely matched that determined experimentally by solution X-ray scattering on ER membranes purified from cells,^36^ and clearly differed from membrane models that include cholesterol and sphingolipids.^37^

**Table 2:** Summary of Simulations Performed in This Study^a^

system	replica	time (μs)/replica	box dimensions (Å)	% water in box	# atoms
TMH1	1	0.5	101 × 101 × 102	62	96,002
	2				
	3				
	4	1.6			
TMH2_5_	1	0.5	102 × 102 × 104	62	99,969
	2				
	3	2.0			
TMH2_6_	1	0.5	112 × 112 × 104	64	96,426
	2				
	3				
	4	2.0			
TMH2_7_	1	1.8	102 × 102 × 103	62	95,431
	2	0.5			
	3	2.0			
TMH2_6_R	1	0.5	102 × 102 × 108	62	1,18,035
	2				
	3				
TMH2_6_L	1	0.5	102 × 102 × 108	64	1,01,967
	2				
	3				
TMH1+amantadine	1	0.20	101 × 101 × 102	62	96,422
TMH2_6_+amantadine	1		105 × 105 × 107	64	96,846
TMH1+verapamil	1		101 × 101 × 93	56	96,638
TMH2_6_+verapamil	1		96 × 96 × 101	58	97,349

a Values are reported for each replica of the indicated systems over the simulation time (μs). Box dimensions refer to the periodic simulation box size for each system (in Å). %Water in box represents the percentage of the total simulation box volume occupied by water, given the average width of the membrane is ∼39 Å.

Each protein model was inserted into its respective membrane, and lipids in steric conflict with the protein were removed. Default protonation states were assigned to ionizable residues, supported by calculations using PropKa.^38^ Prior to membrane insertion, the protein was aligned along the bilayer normal. Lipid molecules with atoms within 1.2 Å of any protein atom were deleted.

The systems were subsequently solvated with water and neutralized with either 150 mM NaCl, 150 mM KCl, or, in some cases, a mixture of KCl and NaCl at 75 mM each. Water molecules overlapping with protein, lipids, or ions (distance <1.2 Å) were removed, yielding the final system sizes reported in Table 2.

#### MD Simulations

All MD simulations were performed using GROMACS (versions 2022.2 or 2023.1).^39,40^ The CHARMM36 force field^35^ was used to model proteins and lipids, with standard CHARMM parameters applied to ions.^41^ Water molecules were described using the TIP3P model.^42^ Each system was first energy minimized using the steepest descent algorithm to remove unfavorable contacts, with a maximum force tolerance of approximately 1000 kJ mol^–1^ nm^–1^.

Equilibration was carried out using a multistage, restrained protocol generated by CHARMM-GUI, with position restraints gradually released on the protein backbone, protein side chains, lipid tail dihedrals, and lipid phosphate headgroups. The equilibration consisted of six sequential runs: two canonical (NVT) simulations of 0.125 ns each, followed by four semi-isotropic isothermal–isobaric (NPT) simulations of 0.125, 0.5, 0.5, and 0.5 ns. During these stages, restraint force constants (kJ mol^–1^ nm^–2^) were progressively reduced as follows: protein backbone from 4000 to 50, protein side chains from 2000 to 0, lipid tail dihedrals from 1000 to 0, and lipid phosphate headgroups from 1000 to 0. All restraints were released in the final equilibration stage before production simulations.

A time step of 1 fs was used for the first three equilibration stages, followed by a 2 fs time step for the remaining equilibration runs. Long-range electrostatic interactions were treated using the smooth particle mesh Ewald (PME) method, with a real-space cutoff of 12 Å and a Fourier grid spacing of 1.4 Å. Lennard–Jones interactions were smoothly switched between 10 and 12 Å.^43,44^ A Verlet neighbor list was employed with a pair-list cutoff of 12 Å, updated every 20 steps.^40^ Bond lengths involving hydrogen atoms in the solute were constrained using the LINCS algorithm,^45^ while water geometry was constrained using the SETTLE algorithm,^46^ allowing the use of a 2 fs integration time step. Equations of motion were integrated using the leapfrog algorithm.^39,40^

Temperature was maintained at 303.15 K using the velocity-rescale (V-rescale) thermostat^47^ with a coupling constant of 1.0 ps. During equilibration, pressure was maintained at 1 atm using a semi-isotropic C-rescale barostat^48^ with a damping constant of 5 ps.

A summary of the simulations carried out in this study is provided in Table 2. Each simulated system is listed along with the number of replicas used and the simulation time per replica (in μs). Additionally, the dimensions of the simulation box (in Å) are reported, along with the percentage of water in the box and the total number of atoms present. The composition of the system is further detailed by providing the percentage of water molecules relative to the total number of atoms included in each simulation.

Analysis of MD trajectories was performed using MDAnalysis^49^ as well as the MDTraj Python package.^50^ Pore dimensions were characterized from the MD trajectories by calculating radius profiles along the putative channel axis using the HOLE plugin^51^ implemented in MDAnalysis.^49^

#### Molecular Docking

The TMH1 and TMH2_6_ hexamer models were selected for molecular docking studies to assess the potential ligandability of the blockers amantadine and verapamil, which have been reported to inhibit viral ion channels and p7-like viroporins using AutoDock Vina.^52^ The starting structures were selected from the equilibrated MD simulations of the respective protein models described in the Results section, with TMH1 representing the extended TMH monomer hexamer that forms large pores and TMH2_6_ being the variant with high pLDDT confidence scores that displayed formation of narrower pores. Prior to docking, protein structures were prepared by removing all water molecules and ions, followed by the addition of polar hydrogen atoms and assignment of Gasteiger partial charges using AutoDockTools for compatibility with AutoDock Vina. Amantadine and verapamil geometries were obtained from the PubChem3D database,^53^ where they were optimized by using the Merck molecular force field (MMFF).^54^

Docking was performed using a grid box encompassing the entire protein, ensuring that all potential binding sites were accessible. AutoDock Vina default parameters were used, including an exhaustiveness of eight, a maximum of 100 binding modes reported per ligand, and an energy range of 3 kcal/mol between the best- and worst-scoring poses. The Vina empirical scoring function was employed, accounting for steric, hydrogen-bonding, hydrophobic, and torsional interactions. The resulting binding poses were ranked according to their Vina scores.

To select nonredundant binding locations, top-scoring poses were filtered based on spatial overlap. Specifically, if poses ranked 1, 2, and 3 overlapped in three-dimensional space, then only rank 1 was retained. If poses ranked 4 and 5 overlapped, then rank 4 was selected. Any subsequent poses (*e.g*., ranks 6 or 7) that overlapped with any previously selected pose were discarded. This procedure was continued until all 100 generated poses were evaluated. Using this approach, 9 and 15 solutions were selected for verapamil and amantadine, respectively, interacting with TMH1, and 13 and 15 solutions were selected for verapamil and amantadine simulations with TMH2_6_, respectively.

All selected docking poses for a given ligand (verapamil or amantadine) with a given model (TMH1 or TMH2_6_) were included in a single simulation system. Each ligand was positioned according to its docking solution, and the poses were carefully checked to ensure they would not perturb one another; that is, ligand positions were independent and did not influence each other artificially. Embedding all poses in a single system allowed us to maintain a consistent protein–membrane environment while reducing computational cost compared with running multiple independent simulations. The protein–ligand complex was embedded in a lipid membrane of the composition described earlier and subjected to MD simulations using the same system preparation workflow and simulation parameters described in the MD section.

Force-field parameters for amantadine and verapamil were automatically derived using CHARMM-GUI with the CHARMM General Force Field (CGenFF)^41^ and were of sufficient quality to be used directly, fully compatible with the CHARMM36 parameters for proteins and lipids, without further modification. All simulations were performed using GROMACS.^39,40^

## Results

### Assembly of *In Silico* Systems Mimicking the Functional Environment of CSFV p7

When assayed experimentally, CSFV p7 displays an ion-channel activity in ER-like lipid bilayers (ER-LBs) characterized by conductance increments of Δγ ∼ 0.1 nS that are compatible with pore radii in the range of *r* ∼ 5–10 Å.^13,21,55^ Figure 1a displays permeability experiments carried out on single ER-like vesicles treated with p7, which revealed that access to their interior depended on solute size. Figure 1b compares pore sizes inferred from these single-vesicle assays with those derived from electrophysiology experiments. Single vesicles were efficiently permeabilized to solutes with MW ≤ 4 kDa (Stokes’ radius ∼ 14 Å), whereas the fraction of vesicles allowing access to solutes with MW ≥ 10 kDa (Stokes’ radius ∼ 23 Å) decreased sharply. On the other hand, conductive levels of ca. 5 ± 3 and 15 ± 3 pA were observed in ER-LBs treated with p7, which supported the coexistence of two different pore structures with approximate radii ranging between 4 and 8 and 7–15 Å, respectively.^21^ Thus, the latter values were consistent with the size cutoff observed in single-vesicle permeability assays.

**Figure 1:**
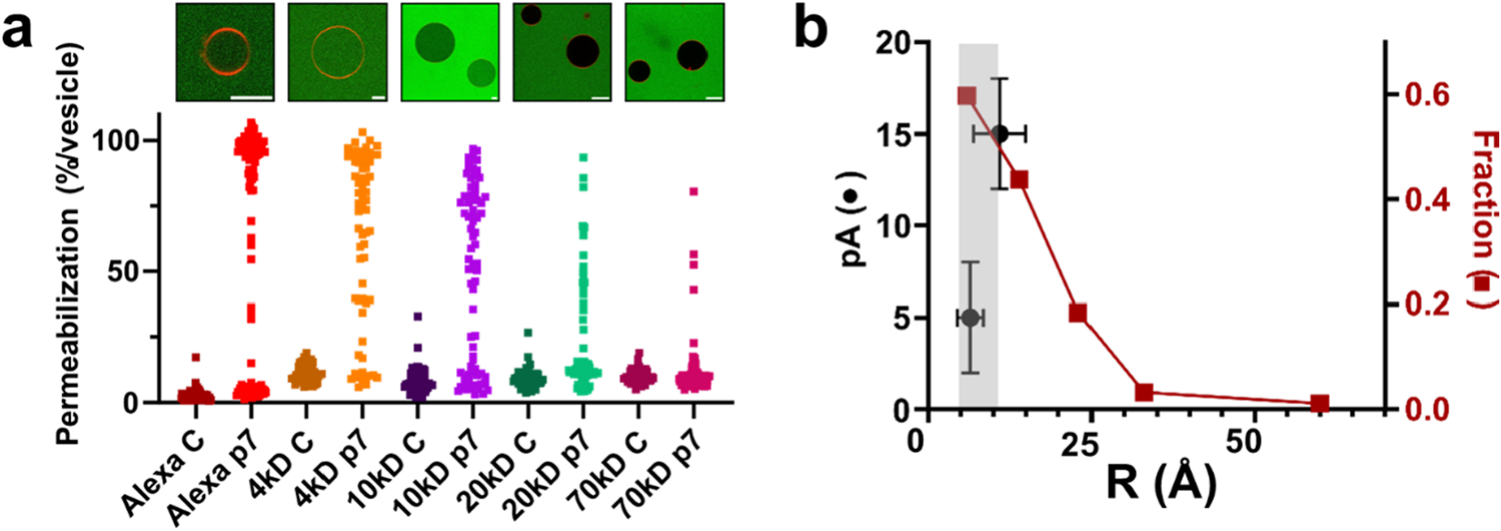
Pore-forming activity of p7 protein experimentally measured in surrogates of the ER membrane. (a) ER-GUV distribution according to the percentage of permeabilization per vesicle measured against solutes of increasing sizes. Samples were treated with p7 (0.2 μM), and percentages of entry into single vesicles calculated for the different solutes after 30 min incubation. Confocal micrographs on top depict selected vesicles comparing degrees of permeabilization to Alexa Fluor 488 and dextrans with increasing MWs. Fluorescence of GUV membranes and solutes are colored in orange and green, respectively. Note that the lipid bilayer integrity is retained in vesicles fully permeabilized to Alexa Fluor or 4 kDa dextrans. Scale bars, 10 μm. (b) Pore size (radius) as a function of the two coexisting ion conductance levels described in ref 21. Radii were estimated from main current jump intensities and applied voltage assuming a cylindrical geometry for the permeating structure (for a detailed description of the procedure followed, please see also ref 50). The permeability levels to solutes of increasing sizes determined in the previous panel are plotted in parallel for comparison. The shadowed area corresponds to the size estimated for conductance increments of Δγ ∼ 120 pS.

To describe this experimental outcome at the molecular level, we performed *in silico* analysis. Previously published evidence supported the capacity of CSFV p7 to form homo-oligomers, including potential hexamers.^52,53^ Consistently, AlphaFold-predicted CSFV p7 hexameric assemblies in which each monomer adopted a compact four-helix bundle arrangement (Figure S1a).

Furthermore, in the constituent monomers, the N-terminal helix h1 was preceded by an extended coil region, in consonance with previous predictions^29^ and experimental determinations indicating that this region of the protein was enriched in extended-chain conformations. In the present study, we used this AlphaFold-derived hexamer as a structural starting point for all of the subsequent *in silico* manipulations and simulations.

The root-mean-square deviation (RMSD) calculated for the hexamer backbone confirmed the stability of this structure when it was inserted in the ER-LB system (Figure S1c). We also established which bundle constituents contributed the most to structural fluctuations in membranes. Across all three replicas, the lowest RMSD was consistently observed for an internal hairpin combining the second and third helices (h2–h3), highlighting the conformational stability of this element when the protein is embedded in the membrane. However, despite the stability displayed by these bundle hexamers, analysis of the pore using the HOLE program^56–58^ did not support an architecture consistent with the ion-channel activity or solute permeabilization observed experimentally in ER-like membranes.

### Stability and Membrane Channel Formation by Extended TMH Hexamer Models Preserving Interprotomer h2–h3 Contacts

We next proceeded to analyze the structural stability and activity of CSFV p7 hexamers containing extended transmembrane hairpins (TMHs) designed to fully span the lipid bilayer (Figures 2, 3, and S2–S4). To generate the hexamers, we inquired about the interactions underpinning the formation and stability of these oligomers. As shown in Figure 2a, formation of the hexamers appeared to involve packing between membrane-embedded helices h2 and h3 of consecutive h2–3 internal hairpins, the protein section that adopted the most stable conformation during the simulations (Figure S1).

**Figure 2:**
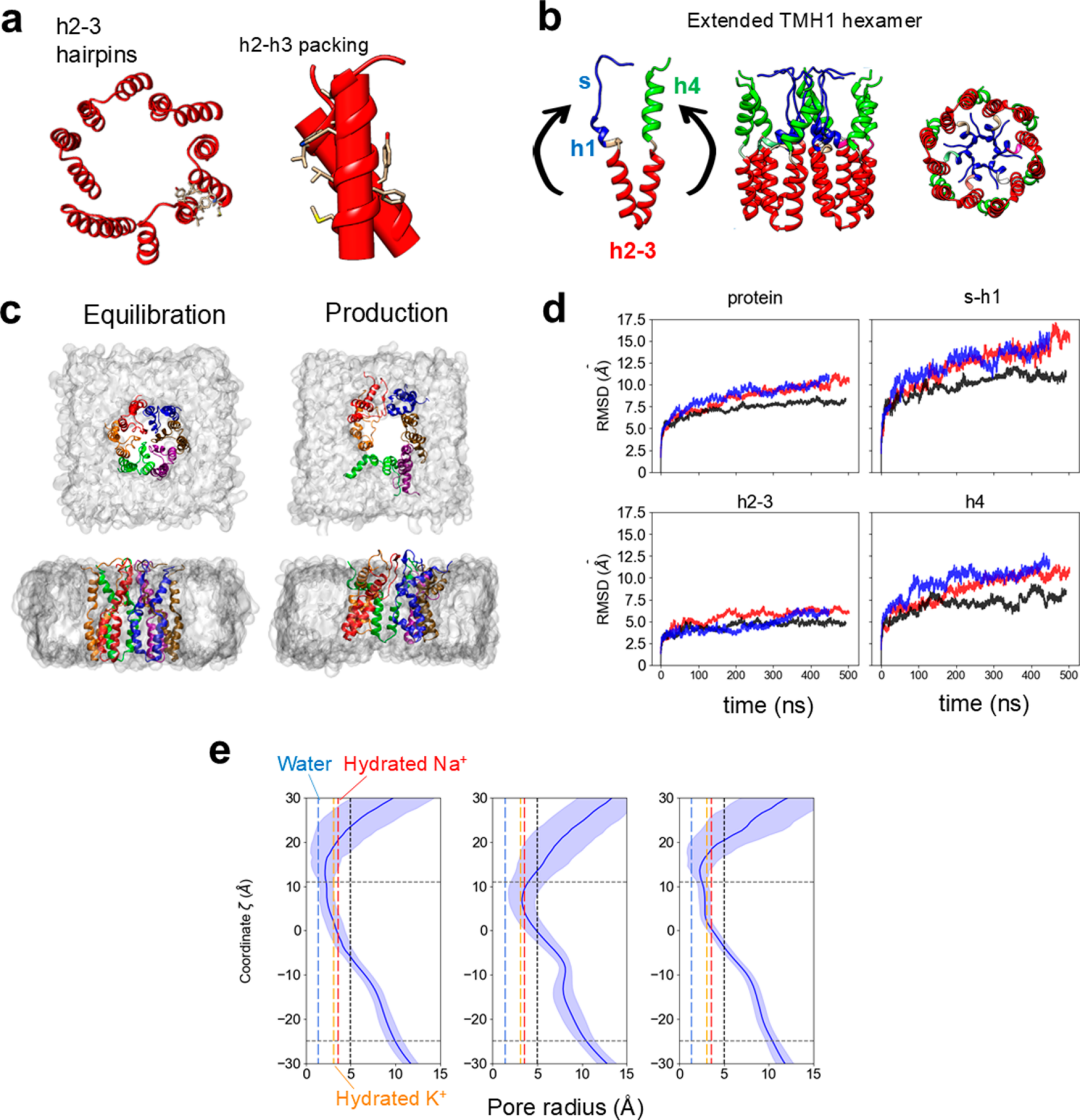
Stability and pore-forming activity of the TMH1 hexamer model in the ER-LB system with NaCl. (a) Helix-helix packing interactions observed between consecutive h2–h3 hairpins at the end of the double-hairpin hexamer/ER-LB system MD simulations (Figure S1). The right panel illustrates the inclination angle between interacting helices 2 and 3 of adjacent hairpins. (b) TMH1 model generation and overall structure. (c) Top and side views of the TMH1 hexamer inserted into the ER-LB. Snapshots were selected after equilibration (left) and after running the simulation (right). (d) RMSD changes during the simulations. Changes over time are shown for the membrane-inserted protein and its different components, *i.e*., s-h1, the h2–h3 hairpin, and h4, as indicated in the panels (see Figure S1a). (e) HOLE-calculated, time-averaged pore radius profiles in three different simulations. Blue, red, and orange dashed lines indicate the radii of water and hydrated Na^+^ and K^+^, respectively. The black dotted line marks a 1 nm diameter pore.

**Figure 3:**
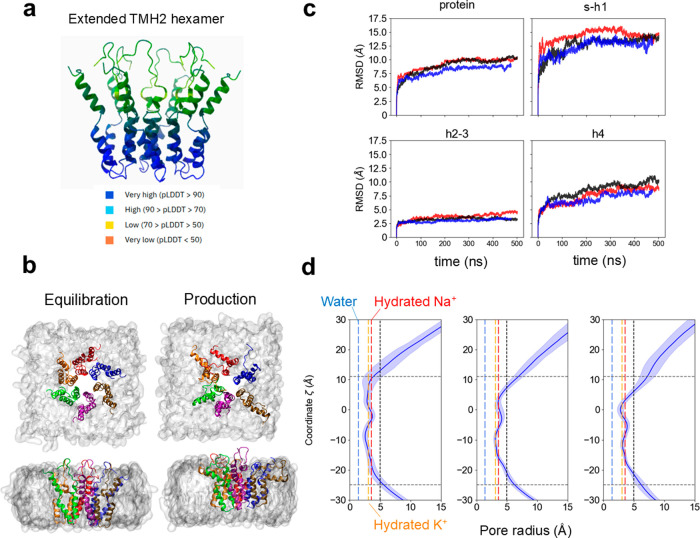
Stability and pore-forming activity of the TMH2_6_ hexamer model in the ER-LB system. (a) TMH2_6_ hexamer model calculated by ColabFold v1.5.5 using the hexamer of extended monomers TMH1 as the starting structure. Blue and green colors denote the high pLDDT of the model (see Figure S4 for a comparison with a low pLDDT model). (b) Top and side views of the TMH2 hexamer inserted into the ER-LB. Snapshots were selected after equilibration (left) and at the end of the simulation (right). (c) RMSD changes occurring during the simulations in the full protein and its different sections. The traces in black, red, and blue correspond to three different replicas. (d) HOLE-calculated, time-averaged pore radius profiles in three different simulations. Blue, orange, and red dashed lines indicate the radii of water, hydrated K^+^, and Na^+^, respectively. The black dotted line marks a 1 nm diameter pore.

The helix crossing-angles (∼40°, right panel) were comparable to those observed in other membrane protein helix-packing motifs.^59^ These interfaces were enriched in hydrophobic residues, including Val24, Tyr25, Leu28, Leu31, Val32, Ile38, Trp41, Ile42, Leu45, and Met49, from h2 and h3, respectively, which remained stably associated throughout the simulations, all of them showing propensity for interhelical contacts.^60^ The packing interactions involving those residues remained essentially unchanged during the simulations of the hexamers in the ER-LB system, thereby underscoring their implication in oligomer assembly and stabilization. These conserved interfacial contacts were therefore used as a structural constraint for building extended oligomeric assemblies.

Using this interaction framework, extended TMH oligomers were generated (Figure 2b). The resulting oligomer model, termed TMH1, portrayed the stable interactions between h2–h3 TMHs observed in the template model, but the constituent monomers fully spanned the lipid bilayer. Moreover, the hexamers assembled wide pores in one bilayer leaflet, stabilized at the base through the lateral interactions between h2–h3 hairpins. Thus, both hairpin helices, h2 and h3, contributed residues that faced the lumen of a wide pore assembled within one bilayer leaflet (*i.e*., h2: Val22, Tyr25, Leu29, Val32-Met33; and h3: Lys39, Leu43, Phe46-His47, Thr50).

MD simulations on the TMH1 model embedded into the ER-LB disclosed the formation of large pores (Figure 2c). Moreover, RMSD changes measured as a function of time revealed a pattern for the conformational fluctuations like those scored for the initial template 3D models, that is, highest for the s-h1 section and lowest for the h2–h3 hairpin (Figure 2d). As stated above, CSFV p7 ion-channel activity in ER-like membranes can display conducting levels characteristic of narrow channels with radii slightly below 4 Å, but also higher levels that denote the presence of larger pore structures (*r* > 5 Å).^21,55^ Further analyses comparing pore hydrations and expansions revealed differences among TMH1–ER–LB systems. In simulations conducted in the presence of K^+^, two of the replicas exhibited substantial pore opening, with channel radii reaching ≥7.5 Å (Table 3 and Figure S2). In contrast, simulations performed with Na^+^ showed more restricted pore dimensions (Table 3 and Figure 2e): only one replica displayed transient pore expansion exceeding 6 Å, whereas the other two rarely achieved radii greater than ∼4 Å. These observations indicate that TMH1 may sample conformational states with enlarged pore geometries under specific conditions, suggesting increased accessibility to hydrated ions, including Ca^2+^. For a comparison, we next tested the feasibility of pore formation using the hairpin model previously proposed by Gladue et al.^29^ In MD simulations, these assemblies rapidly collapsed into closed configurations and failed to form stable, functional membrane channels (Figure S3).

**Table 3:** Summary of Water and Ion Occupancy within the Channel Region^a^

						**mixtures**	
**system**	**replica**	**time (μs) per replica**	**% water column**	**# K** ^**+**^ **permeating**	**# Na** ^**+**^ **permeating**	**# K** ^**+**^ **permeating**	**# Na** ^**+**^ **permeating**
TMH1	1	0.5	99	82 (24)	6 (2)	13 (4)	18 (7)
	2			7 (4)	62 (3)	30 (9)	15 (8)
	3			112 (24)	1	13 (8)	12 (4)
	4	1.6			431(91)		
TMH2_5_(pentamer)	1	0.5	<1		0		
	2		1		0		
	3		<1		0		
TMH2_6_(hexamer)	1	0.5	25		0		
	2		71		1		
	3		82		0		
	4	2.0	0.23		0		
TMH2_7_(heptamer)	1	1.8	100		46 (26)		
	2	0.5			20 (5)		
	3	2	0		0		
TMH2_6_R (reverse of TMH2_6_)	1	0.5	<1		0		
	2				0		
	3				0		
TMH2_6_L	1	0.5	0		0		
	2		<1		0		
	3		1		0		
TMH1+amantadine	1	0.2	97;96^b^	0	1		
TMH2_6_+amantadine	1	0.2	60;5^b^	0	0		
TMH1+verapamil	1	0.2	84;97^b^	3	1		
TMH2_6_+verapamil	1	0.2	9;7^b^	0	0		

a Values are reported for each replica of the indicated systems over the total simulation time (μs). % Water Column refers to the fraction of simulation time during which water molecules are detected within the full channel region, defined as a cylinder with a radius of 6.3 Å. Values are reported separately for each replica when they are not identical. For single-ion systems, the electrolyte concentration was either 150 mM KCl or 150 mM NaCl. For mixed-ion systems, both KCl and NaCl were present at 75 mM each. For replicas in which the model collapsed and the central pore was closed, no permeation of ions was detected, consistent with the absence of a continuous water column. The number of ions crossing the membrane is given for a cylinder of *r* ∼ 24.5 Å and in parentheses if different for *r* = 14 Å.

b Two values are reported for each condition, corresponding to simulations performed with K+ (first) and Na+ (second).

### Membrane Channel Formation by Stable Models Based on Extended TMH Monomers

Although the above-described TMH1 pores appeared to reproduce at the molecular level the experimentally measured large membrane pores induced by p7 (Figure 1), these permeating structures seemed to display limited stability. Therefore, using TMH1 as a template, we sought to produce more stable pore-forming hexamers, termed TMH2_6_, using ColabFold v1.5.5 (Figure 3).

In one of the recovered TMH2_6_ models (Figure 3a), the s-h1-kink-h2 motif occupied a central position, potentially forming a pore. The s-h1 sections assembled an open vestibule, whereas h2-h2 packing interactions, through residues Ile20, Val23, Leu27, Tyr30, Val22, Tyr25, Leu29, Val32 from consecutive helices, contributed to the formation of a narrow pore, which was lined by residues Glu21, Val24, Leu28, Leu31-Val32, and Arg34. As shown in Figure 3b,c, this model appeared to constantly maintain channels in an open state during the simulations and displayed RMSD change patterns consistent with its enhanced stability. Further analysis using the HOLE program confirmed the assembly of pores with radii of approximately 3 Å at the narrowest sections (Figure 3d,e) in the three replicas. Thus, these stable pores allowed passage of water molecules and potentially might enable permeation of hydrated ions.

To establish the structural robustness of this model, we performed similar analyses on TMH2_6_L hexamers rendered with lower pLDDT scores (Figure S4). When embedded in the ER-LB, this model structure displayed higher RMSDs and did not assemble pores. These results indicate that pore formation is strongly dependent on the prediction confidence and structural integrity of the TMH architecture.

Experimental evidence suggests that while CSFV p7 forms detergent-resistant oligomers including hexamers, formation of higher-order oligomers such as heptamers has also been reported for related viral p7 proteins (*e.g*., HCV p7),^4^ although this remains less well defined. Thus, given the small radii of the pores established by TMH2_6_, we next tested the possible effect of incorporating an extra monomer into the oligomer to form a heptamer, TMH2_7_ (Figure 4).

**Figure 4:**
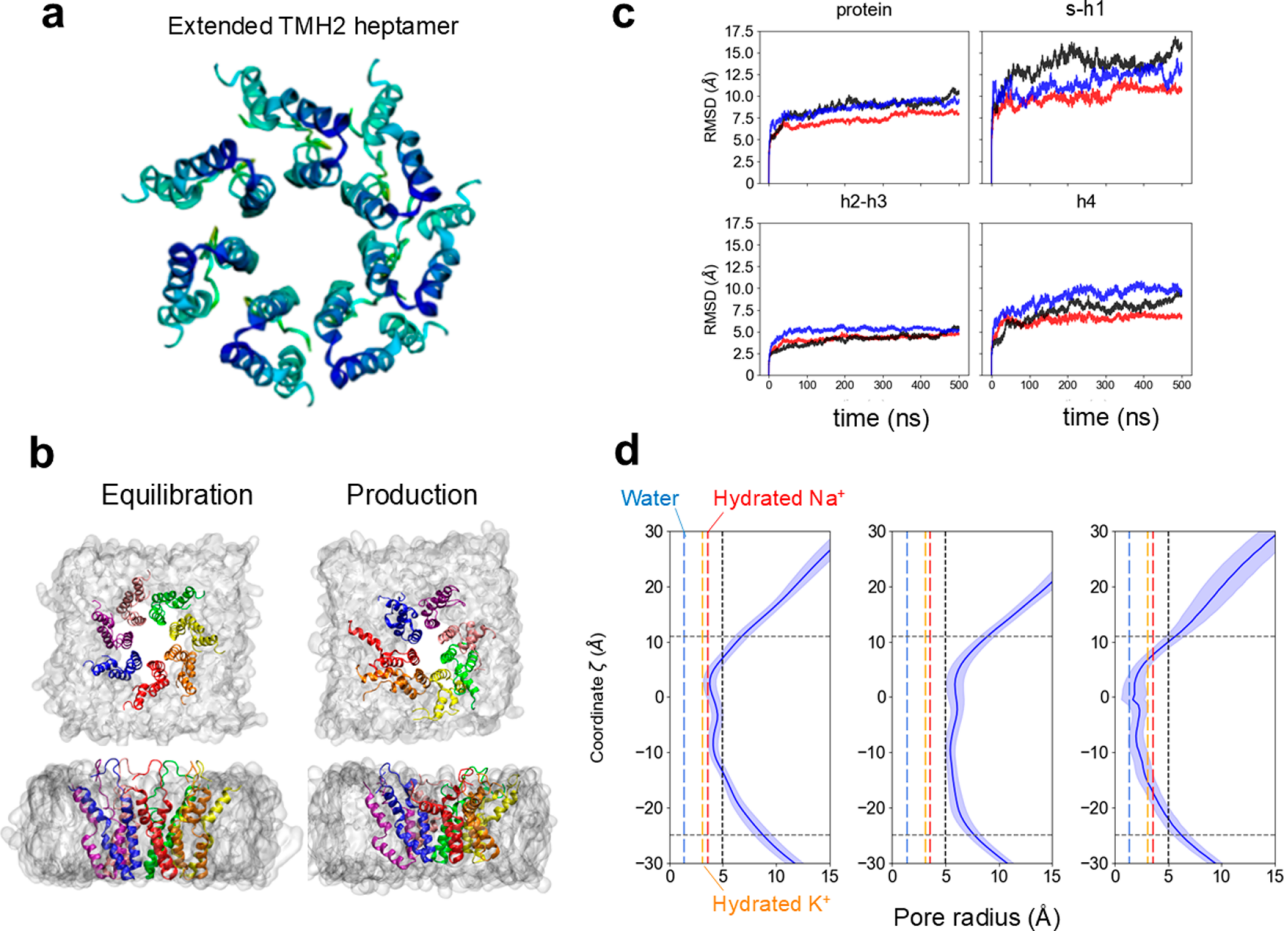
Stability and pore-forming activities of TMH2_7_ in the ER-LB system. (a) TMH2_7_ heptamer model calculated by ColabFold v1.6.1 using TMH1 as the template structure. Blue and green colors denote the high pLDDT of the model. (b) Top and side views of the TMH2 heptamer inserted into the ER-LB. Snapshots were selected after equilibration (left) and at the end of the simulation (right). (c) RMSD changes occurring during the simulations in the full protein and its different sections. The traces in black, red, and blue correspond to three different replicas. (d) HOLE-calculated, time-averaged pore radius profiles in three different simulations. Blue, orange, and red dashed lines indicate the radii of water, hydrated K^+^, and Na^+^, respectively. The black dotted line marks a 1 nm diameter pore.

For TMH2_7_, the results indicated pores sharing key features with TMH1 assemblies, including pore radii between 4 and 6 Å, continuous hydration columns, stable intersubunit packing, and partial ion accessibility under simulation conditions (Figure 4 and Table 3). Sodium was able to traverse this heptamer in the first replicate at bottleneck radii of just under 4 Å (Figure 5).

**Figure 5:**
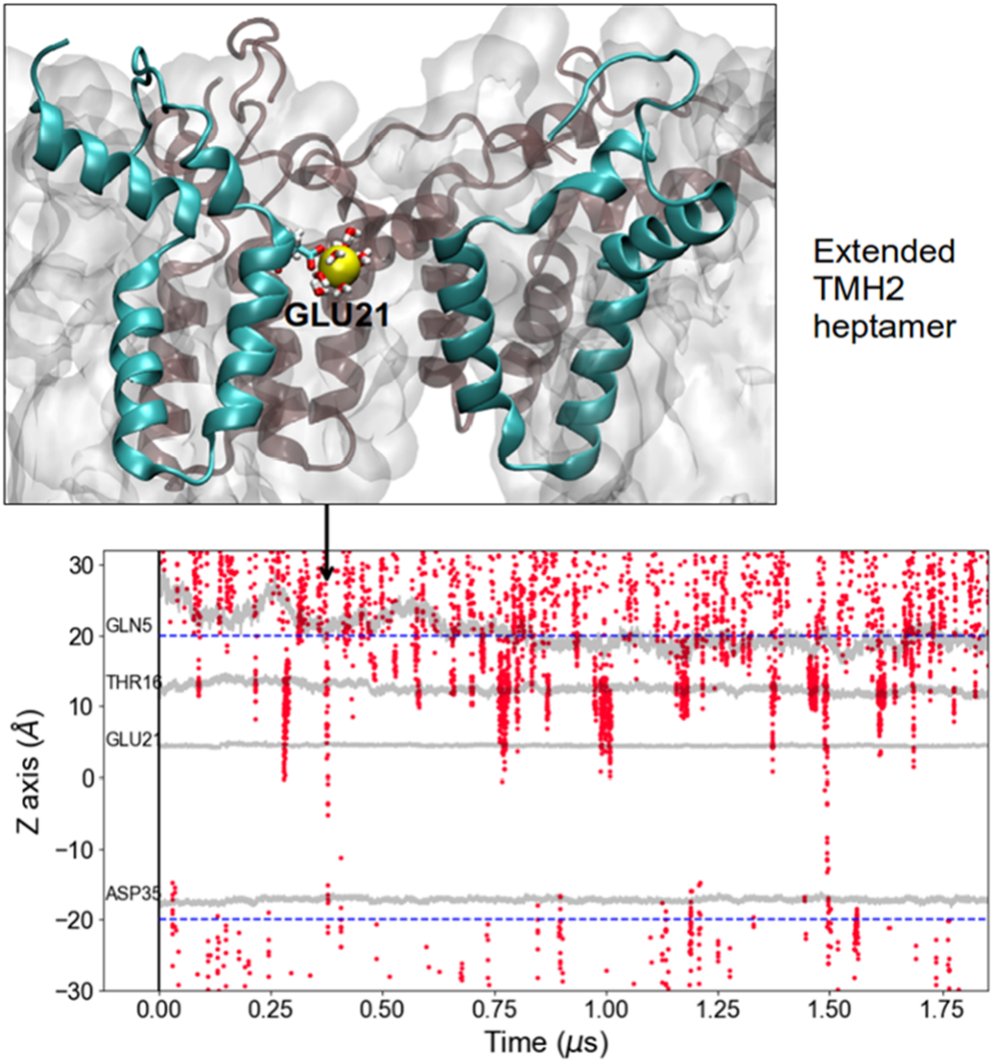
Sodium transport through the predicted heptamer is associated with Glu21 and the hydrophobic residues Ile20, Leu24, and Leu28. Blue dotted lines represent the average positions of the lipid phosphate groups in the extracellular (EC) and intracellular (IC) membrane leaflets. The black arrow indicates the time at which the snapshot was taken. During the 2.5 ns traversal of the sodium ion from the EC to the IC side, the ion was coordinated by Glu21 and subsequently interacted with Ile20, Leu24, and Leu28, while remaining coordinated mainly by water throughout the process.

Transport of this cation through the predicted heptamer was associated with Glu21 and hydrophobic residues Ile20, Leu24, and Leu28. Across simulations, the sodium ion shows a recurring coordination pattern, being initially associated with Glu21 and subsequently interacting with Ile20, Leu24, and Leu28 while remaining predominantly hydrated throughout the trajectory.

To further determine the effect of the oligomer size, we also generated p7 assemblies made of five protomers (TMH2_5_). Figure 6 presents the structure, stability, and pore-forming properties of TMH2_5_, a pentamer model based on extended monomers. Reduction of the number of protomers did not affect overall complex stability in the ER-LB system but resulted in the formation of pores unable to conduct ions (see also Table 3).

**Figure 6:**
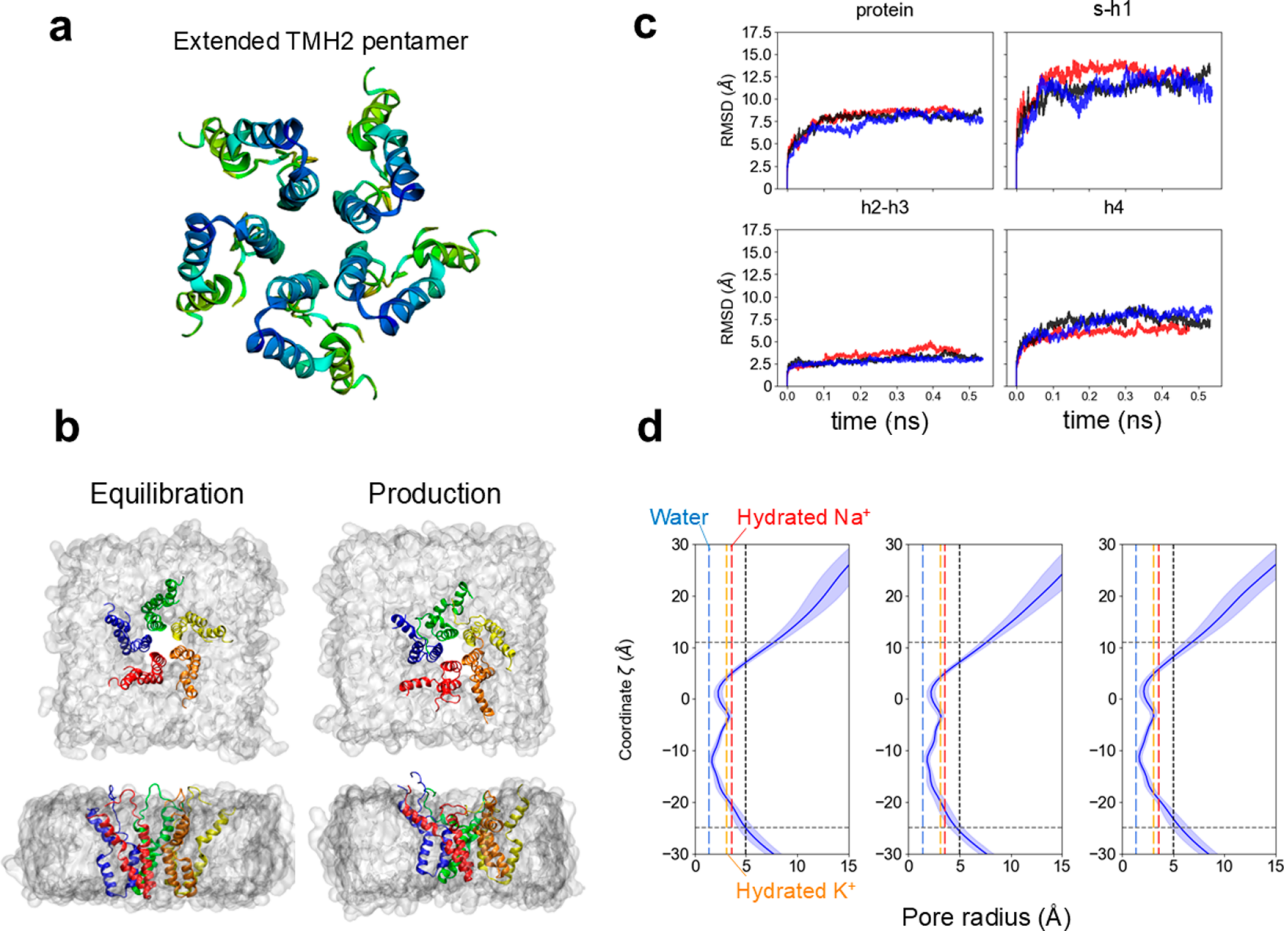
Stability and pore-forming activities of TMH2_5_ in the ER-LB system. (a) TMH2_5_ model calculated by ColabFold v1.6.1 using TMH1 as the template structure. Blue and green colors denote the high pLDDT of the model. (b) Top and side views of the TMH2_5_ pentamer inserted into the ER-LB. Snapshots were selected after equilibration (left) and at the end of the simulation (right). (c) RMSD changes occurring during the simulations in the full protein and its different sections. The traces in black, red, and blue correspond to three different replicas. (d) HOLE-calculated, time-averaged pore radius profiles in three different simulations. Blue, orange, and red dashed lines indicate the radii of water, hydrated K^+^, and Na^+^, respectively. The black dotted line marks a 1 nm diameter pore.

Figure 7 and Table 3 summarize the pore-forming features observed in the different ER-LB-p7 systems. Comparison of water occupancy and ion permeation across the different CSFV p7 assemblies reveals a continuum of pore states ranging from nonconductive, collapsed conformations to partially and fully hydrated assemblies (Table 3). TMH1 and TMH2_7_ exhibited high water occupancy (approximately 100% in most replicas), consistent with the formation of stable hydrated pore-like architectures supporting intermittent ion permeation. In contrast, TMH2_6_ showed intermediate and more variable hydration (25–82%), suggesting a metastable state in which pore formation is only partially maintained over simulation time scales. TMH2_5_ pentamers and the low-confidence or alternative conformations (TMH2_6_L, TMH2_6_R) remained essentially dehydrated (<1% water occupancy), consistent with collapsed or nonconductive assemblies under the present simulation conditions.

**Figure 7:**
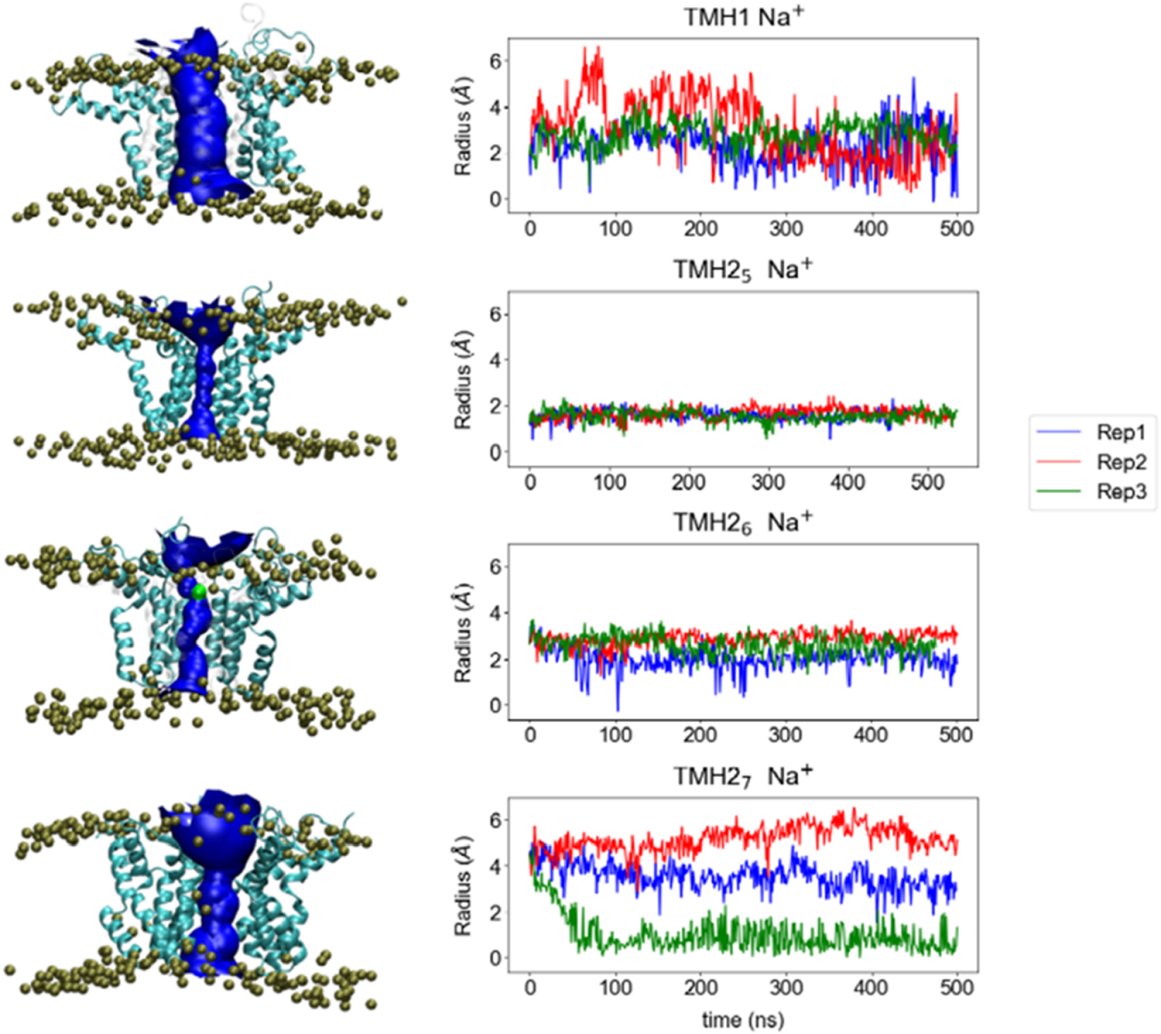
Pore surface diagrams and evolution of the radii over simulation times (left and right panels, respectively). Pore radius profiles correspond to the minimum radius along the pore axis, as calculated using HOLE. Individual replicas are shown in different colors for each system. Replicates extending to 2 μs were truncated to 0.5 μs to match the simulation time of the other replicas.

In systems where ion permeation was observed, local electrostatic contributions from Glu21 may further facilitate transient ion stabilization within the constriction zone, in combination with the local hydration environment and pore geometry defined by surrounding residues that define the pore architecture (Figure 5).

Ion permeation events broadly followed this hydration hierarchy. TMH1 supported frequent K^+^ and Na^+^ permeation events across replicas, whereas TMH2_7_ exhibited more limited and replica-dependent Na^+^ permeation. TMH2_6_ displayed only sparse ion passage, and TMH2_5_ as well as the low-hydration models showed negligible or no permeation within the simulation time scale.

Taken together, these results suggest that sustained pore hydration is a key determinant of ion accessibility in these models, whereas oligomeric state alone does not reliably predict conductive behavior. Instead, conduction appears to emerge from a coupled dependence on both the structural stability of the transmembrane bundle and the persistence of a continuous hydration column.

### Interactions of Extended TMH Models with Channel Blockers

To extend the structural analysis of the different CSFV p7 assemblies, we next investigated their potential interactions with known channel blockers, focusing on the hexameric assembly, which is most consistent with our experimentally observed conductive states in ER-like lipid bilayers.

It should be emphasized that the analyses presented in this section are exploratory and aim to investigate potential ligand-binding modes within structurally stable p7 hexamer assemblies. The docking and molecular dynamics simulations do not include free-energy calculations or electrophysiological constraints and therefore cannot be used to infer binding affinities, specificity, or functional inhibition of ion conductance. Instead, these simulations provide qualitative insight into possible ligand–pore interactions and their structural consequences, such as changes in pore hydration and steric occupancy, within the hexameric p7 model.

Previous experimental studies have shown that both amantadine and verapamil reduce CSFV infectivity and inhibit p7-mediated membrane permeabilization in model lipid systems,^22,29^ supporting their roles as functional channel blockers. However, to the best of our knowledge, no structural or biophysical data are currently available describing their direct binding mode to CSFV p7, nor is there evidence defining the binding stoichiometry or potential cooperative interactions. In this context, the present simulations aim to provide a molecular-level hypothesis for possible binding configurations and their functional consequences on the channel conductance.

The structural stability and channel assembly of the TMH1 and TMH2_6_ models support CSFV p7 architectures in ER-like membranes distinct from those anticipated by earlier predictions. To evaluate potential interactions with known channel blockers, docking studies were performed using the top-scoring poses filtered for nonredundant binding locations, as described above. Figure 8 shows representative poses for verapamil, a clinically used calcium channel blocker, and amantadine, an antiviral drug known to inhibit viral ion channels, at the start of the simulations and after ∼200 ns in the ER-LB system.

**Figure 8:**
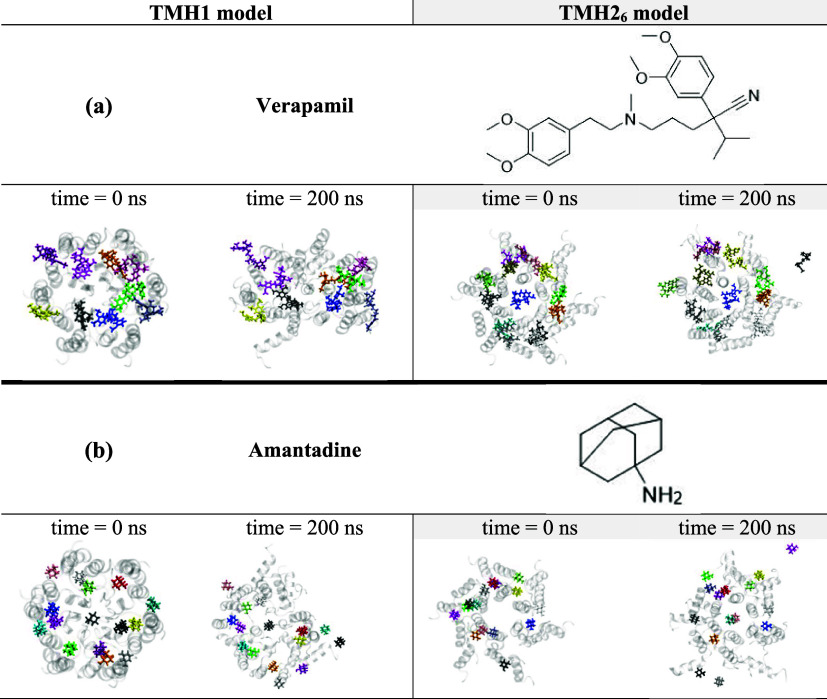
Interactions of channel blockers with TMH1 and TMH2_6_ hexamers in membranes. Docking results for (a) verapamil and (b) amantadine are shown at the start of the simulation and after 200 ns for the complexes in the ER-LB. Each molecule is depicted in a distinct color, while the protein is shown in gray using a cartoon representation, viewed perpendicular to the membrane plane.

Docking of amantadine or verapamil reduced water passage, particularly in TMH2_6_, with TMH2_6_+verapamil showing almost complete inhibition, whereas TMH1 largely retained hydration and partial ion flow. At the end of the trajectories, verapamil is found to block the pore center in TMH2_6_, interacting with residues Glu21 and Ala24 and suggesting direct inhibition of channel conduction (Figure 8a). In TMH1, amantadine was positioned at the pore center while verapamil remained at the periphery. By the end of the simulations, both ligands were stably associated with the central pore in their respective TMH hexamers. These observations suggest that distinct TMH assemblies may differ in their accessibility to small-molecule ligands and in the structural consequences of ligand binding.

## Discussion

The “ion channel-pore dualism” or “unconventional behavior” observed in ion channels appears to characterize the membrane activity of many members of the viroporin family.^3,11,14^ The fact that, in contrast to the sophisticated and complex built-in architectures of “classical” ion channels, viroporin channels may be constrained by the surrounding membrane, has been suggested to explain this unconventional activity.^3^ However, structural evidence supporting this assumption is still lacking in the viroporin field. In this study, we have focused our attention on the structure–function of CSFV p7, an example of an unconventional ion channel, and described its stability and pore formation dynamics in membranes based on AlphaFold hexameric models. We used those models as templates, first for generating extended monomeric hairpins, capable of spanning fully the ER-like lipid bilayer, and then to assemble these monomers in the form of membrane-inserted oligomers. These newly generated models initially retained two basic structural features predicted by AlphaFold, namely: a hexameric quaternary structure and the conformation of the h2–h3 hairpin, the p7 element showing highest pLDDT scores and stability during the simulations. We also examined the effect of adding or removing protomers on the stability and pore-forming capacity of the membrane-inserted p7.

Considering hydrated K^+^ and Ca^2+^, the two cations whose homeostasis is mainly altered through viroporin activity in cells,^1,4,8^ the TMH2_6_ hexamer sampled stable conductive states during simulations. However, these TMH2_6_ pores were narrower than the p7 pores determined experimentally. In comparison, TMH2_7_ heptamers and TMH1 hexamers formed pore geometries with dimensions compatible with the passage of hydrated ions and small solutes.^13,21^ TMH1, TMH2_6_, and TMH2_7_ collectively reproduce different aspects of experimentally observed p7 permeability, spanning narrower and wider conductive states. All these models sampled conductive conformations, with TMH2_6_ favoring narrower stable pores and TMH1/TMH2_7_ exploring wider pore geometries. Rather than discrete and stable pore types, our results are also consistent with a dynamic ensemble of oligomeric and lipid-influenced conductive states, in agreement with the unconventional ion-channel behavior described for viroporins.

Our MD simulation analyses in the ER-LB system may provide clues for understanding, at the molecular level, the basis of the unconventional behavior of the CSFV p7 viroporin. The barrel-stave organization of the TMH2_6_ model was overall preserved during the simulations, and it reproduced features of conventional channels, most prominently: (i) a hydrated and ion-accessible state with a defined size, large enough to allow permeation of Na^+^ and K^+^ ions; and (ii) binding of verapamil via a standard pore-blocking mechanism. Together, these observations imply that TMH2_6_-like assemblies may represent a structurally defined and druggable conformational state of p7.

In comparison, membrane pores established by TMH1 and TMH2_7_ models exhibited large sizes, demonstrating their potential to conduct Ca^2+^ ions and larger solutes (Figure 7). These wide pores may be less amenable to blockade by conventional pore-occluding inhibitors. The observed effect of verapamil suggests that inhibitors of protein–protein interactions may exert greater effects by disrupting interoligomer interactions and, in turn, the quaternary structure required for solute permeation.

We emphasize that, even though the structural models described in this work support the possibility of p7 adopting alternative membrane channel architectures, our simulations do not provide potential pathways for the structural transition from one form to another. From the perspective of conformational stability, the three models, TMH1, TMH2_6_, and TMH2_7_, appear to represent likely forms of p7 inserted into the ER after biogenesis. Moreover, we note that our simulations did not reveal p7 pores with radii exceeding 10 Å, which might be responsible for the observed residual GUV permeability to large dextrans (Figure 1a). These structures, potentially relevant for the progression of cell infection, might involve an increased number of protomers and/or be proteolipidic in nature, as described for other unconventional viroporins that form large pores.^3^ At any rate, the fact that, after full permeabilization, the integrity of the membrane was maintained in the vesicles would not be consistent with the involvement of nonspecific mechanisms disrupting the lipid bilayer architecture. Thus, p7 folding into a particular form could depend on unknown factors evolving during the replicative cycle of the virus, including alterations in the ER membrane potential or lipid nanoenvironment and changes in interactions with other viral or cellular proteins.

Importantly, these findings highlight the functional versatility of the p7 protein, suggesting that its role in viral physiology may extend beyond that of a simple ion channel. The existence of multiple stable conformations could allow p7 to adapt dynamically to different cellular environments or stages of the viral life cycle, potentially regulating ion fluxes and protein interactions in a context-dependent manner. This flexibility may also explain why p7 has been difficult to inhibit effectively by using standard small-molecule blockers and underscores the need for alternative therapeutic strategies.

## Conclusions

The experimental characterization of the p7 structures remains extremely challenging. As a small, highly hydrophobic membrane protein, p7 is difficult to crystallize or study using standard NMR approaches, and its structural heterogeneity further complicates such efforts. In this context, our simulations provide a valuable window into possible p7 conformations, offering atomistic insights that are difficult to obtain by experimental methods alone. While free-energy calculations and applied field simulations would be required to quantitatively estimate ion permeation barriers and conductances, the present study has focused on structural stability, pore hydration, and comparative oligomeric behavior, providing a qualitative structural framework for future thermodynamic investigations.

Our results further provide a framework for future experimental studies aimed at dissecting the structure–function relationships of p7. High-resolution structural techniques, combined with mutational analysis and functional assays, could validate the predicted channel architectures and clarify the molecular determinants governing the formation of TMH1/TMH2_7_ versus TMH2_6_-like pores. In this regard, the potential application of low-resolution structural techniques as the substituted-cysteine accessibility method (SCAM)^61,62^ or the cross-linking mass spectrometry (XL-MS),^63^ may additionally offer viable alternatives to the study of p7 structure–function relationships. Ultimately, understanding these determinants may not only inform the design of targeted antivirals but also shed light on the broader principles underlying the behavior of viral viroporins in host membranes.

## Supplementary Material



## Notes

Summary of CSFV p7 oligomeric models generated in this study, including predicted confidence metrics (pLDDT, PAE, pTM, and ipTM scores) and PAE plots; generation and conformational stability of extended monomers inserted into membranes; stability and pore-forming activity of TMH1 hexamers with KCl; MD simulations of previously proposed CSFV p7 models; and stability and pore-forming activity of TMH2_6_L hexamers with low pLDDT scores (Table S1 and Figures S1–S4) (PDF)

## Abbreviations Used

CSFV: classical swine fever virus
ER-LB: endoplasmic reticulum-like lipid bilayer
GUV: giant unilamellar vesicle
POPC: 1-palmitoyl-2-oleoyl-glycero-3-phosphocholine
POPE: 1-palmitoyl-2-oleoyl-glycero-3-phosphoethanolamine
POPI: 1-palmitoyl-2-oleoyl-glycero-3-phosphoinositol
TMH: transmembrane hairpin

## References

[1] Nieva J. L., Madan V., Carrasco L. (2012). Viroporins:
structure and biological
functions. Nat. Rev. Microbiol..

[2] Devantier K., Kjaer V. M. S., Griffin S., Kragelund B. B., Rosenkilde M. M. (2024). Advancing the field of viroporins-Structure,
function
and pharmacology: IUPHAR Review X. Br. J. Pharmacol..

[3] Alcaraz A., Nieva J. L. (2025). Viroporins: discovery, methods of study, and mechanisms
of host-membrane permeabilization. Q. Rev. Biophys..

[4] Scott C., Griffin S. (2015). Viroporins: structure, function and
potential as antiviral
targets. J. Gen. Virol..

[5] To J., Surya W., Torres J. (2016). Targeting
the Channel Activity of
Viroporins. Adv. Protein Chem. Struct. Biol..

[6] Breitinger U., Farag N. S., Sticht H., Breitinger H. G. (2022). Viroporins:
Structure, function, and their role in the life cycle of SARS-CoV-2. Int. J. Biochem. Cell Biol..

[7] DiMaio D. (2014). Viral miniproteins. Annu. Rev. Microbiol..

[8] Hyser J. M., Estes M. K. (2015). Pathophysiological
Consequences of Calcium-Conducting
Viroporins. Annu. Rev. Virol..

[9] Nieto-Torres J. L., Verdia-Baguena C., Castano-Rodriguez C., Aguilella V. M., Enjuanes L. (2015). Relevance of Viroporin
Ion Channel Activity on Viral
Replication and Pathogenesis. Viruses.

[10] Delcour, A. H. Electrophysiology of Unconventional Channels and Pores; Springer, 2015; 10.1007/978-3-319-20149-8.

[11] Hyser, J. M. Viroporins. In Electrophysiology of Unconventional Channels and Pores; Delcour, A. H. , Ed.; Springer, 2015; pp 153–181.

[12] Montserret R., Saint N., Vanbelle C., Salvay A. G., Simorre J. P., Ebel C., Sapay N., Renisio J. G., Bockmann A., Steinmann E. (2010). NMR structure and ion channel activity of the
p7 protein from hepatitis C virus. J. Biol.
Chem..

[13] Largo E., Queralt-Martín M., Carravilla P., Nieva J. L., Alcaraz A. (2021). Single-molecule conformational dynamics
of viroporin ion channels regulated by lipid-protein interactions. Bioelectrochemistry.

[14] Mehnert T., Routh A., Judge P. J., Lam Y. H., Fischer D., Watts A., Fischer W. B. (2008). Biophysical
characterization of Vpu
from HIV-1 suggests a channel-pore dualism. Proteins:Struct., Funct., Bioinf..

[15] Saffarian
Delkhosh A., Hadadianpour E., Islam M. M., Georgieva E. R. (2025). Highly
versatile small virus-encoded proteins in cellular membranes: A structural
perspective on how proteins’ inherent conformational plasticity
couples with host membranes’ properties to control cellular
processes. J. Struct. Biol.:X.

[16] Acharya R., Carnevale V., Fiorin G., Levine B. G., Polishchuk A. L., Balannik V., Samish I., Lamb R. A., Pinto L. H., DeGrado W. F., Klein M. L. (2010). Structure and mechanism of proton
transport through the transmembrane tetrameric M2 protein bundle of
the influenza A virus. Proc. Natl. Acad. Sci.
U. S. A..

[17] Sharma M., Yi M., Dong H., Qin H., Peterson E., Busath D. D., Zhou H. X., Cross T. A. (2010). Insight into the mechanism of the
influenza A proton channel from a structure in a lipid bilayer. Science.

[18] Surya W., Li Y., Torres J. (2018). Structural
model of the SARS coronavirus E channel
in LMPG micelles. Biochim. Biophys. Acta, Biomembr..

[19] Mandala V. S., McKay M. J., Shcherbakov A. A., Dregni A. J., Kolocouris A., Hong M. (2020). Structure and drug binding of the SARS-CoV-2 envelope protein transmembrane
domain in lipid bilayers. Nat. Struct. Mol.
Biol..

[20] Medeiros-Silva J., Somberg N. H., Wang H. K., McKay M. J., Mandala V. S., Dregni A. J., Hong M. (2022). pH- and Calcium-Dependent
Aromatic
Network in the SARS-CoV-2 Envelope Protein. J. Am. Chem. Soc..

[21] Largo E., Verdia-Baguena C., Aguilella V. M., Nieva J. L., Alcaraz A. (2016). Ion channel
activity of the CSFV p7 viroporin in surrogates of the ER lipid bilayer. Biochim. Biophys. Acta.

[22] Largo E., Gladue D. P., Huarte N., Borca M. V., Nieva J. L. (2014). Pore-forming
activity of pestivirus p7 in a minimal model system supports genus-specific
viroporin function. Antiviral Res..

[23] Jumper J., Evans R., Pritzel A., Green T., Figurnov M., Ronneberger O., Tunyasuvunakool K., Bates R., Žídek A., Potapenko A. (2021). Highly accurate protein structure prediction
with AlphaFold. Nature.

[24] Mirdita M., Schütze K., Moriwaki Y., Heo L., Ovchinnikov S., Steinegger M. (2022). ColabFold: making protein folding accessible to all. Nat. Methods.

[25] Chen X., Wang X. (2024). Computational investigation in inhibitory
effects of amantadine on
classical swine fever virus p7 ion channel activity. Sci. Rep.

[26] Suhag K., Borkotoky S., Siddiqui S. I., Kumar J., Kumar C. S., Tatiya P., Ghosh S., Banerjee M. (2025). Mechanistic Insights
into the Divergent Membrane Activities of a Viroporin from Chikungunya
Virus and Its Transframe Variant. ACS Infect.
Dis..

[27] Devantier K., Toft-Bertelsen T. L., Prestel A., Kjær V. M. S., Sahin C., Giulini M., Louka S., Spiess K., Manandhar A., Qvortrup K. (2025). The SH protein of mumps
virus is a druggable pentameric viroporin. Sci.
Adv..

[28] Surya W., Goh J., Ponniah C., Torres J. (2025). AlphaFold Prediction of Protein-Protein
Interactions in the Flaviviridae Proteomes. Int. J. Mol. Sci..

[29] Gladue D. P., Holinka L. G., Largo E., Fernandez Sainz I., Carrillo C., O’Donnell V., Baker-Branstetter R., Lu Z., Ambroggio X., Risatti G. R. (2012). Classical swine fever
virus p7 protein is a viroporin involved in virulence in swine. J. Virol..

[30] Gladue D. P., Gomez-Lucas L., Largo E., Ramirez-Medina E., Torralba J., Queralt-Martín M., Alcaraz A., Velazquez-Salinas L., Nieva J. L., Borca M. V. (2024). Viroporin-like
activity
of the hairpin transmembrane domain of African swine fever virus B169L
protein. J. Virol..

[31] Gladue D. P., Largo E., de la Arada I., Aguilella V. M., Alcaraz A., Arrondo J. L. R., Holinka L. G., Brocchi E., Ramirez-Medina E., Vuono E. A. (2018). Molecular
Characterization
of the Viroporin Function of Foot-and-Mouth Disease Virus Nonstructural
Protein 2B. J. Virol..

[32] Finn R. D., Clements J., Eddy S. R. (2011). HMMER web server:
interactive sequence
similarity searching. Nucleic Acids Res..

[33] Holthuis J. C. M., Menon A. K. (2014). Lipid landscapes and pipelines in membrane homeostasis. Nature.

[34] Feng S., Park S., Choi Y. K., Im W. (2023). CHARMM-GUI Membrane
Builder: Past, Current, and Future Developments and Applications. J. Chem. Theory Comput..

[35] van
Meer G., Voelker D. R., Feigenson G. W. (2008). Membrane lipids: where they are and
how they behave. Nat. Rev. Mol. Cell Biol..

[36] Mitra K., Ubarretxena-Belandia I., Taguchi T., Warren G., Engelman D. M. (2004). Modulation
of the bilayer thickness of exocytic pathway membranes by membrane
proteins rather than cholesterol. Proc. Natl.
Acad. Sci. U. S. A..

[37] Domene C., Wiley B., Insausti S., Rujas E., Nieva J. L. (2025). Distinctive
Membrane Accommodation Traits Underpinning the Neutralization Activity
of HIV-1 Antibody against MPER. Mol. Pharmaceutics.

[38] Olsson M. H. M., Søndergaard C. R., Rostkowski M., Jensen J. H. (2011). PROPKA3: Consistent Treatment of Internal and Surface
Residues in Empirical pKa Predictions. J. Chem.
Theory Comput..

[39] Van
Der Spoel D., Lindahl E., Hess B., Groenhof G., Mark A. E., Berendsen H. J. (2005). GROMACS: fast, flexible, and free. J. Comput. Chem..

[40] Abraham M. J., Murtola T., Schulz R., Páll S., Smith J. C., Hess B., Lindahl E. (2015). GROMACS: High
performance
molecular simulations through multi-level parallelism from laptops
to supercomputers. SoftwareX.

[41] Vanommeslaeghe K., Hatcher E., Acharya C., Kundu S., Zhong S., Shim J., Darian E., Guvench O., Lopes P., Vorobyov I., Mackerell A. (2010). CHARMM general
force field: A force
field for drug-like molecules compatible with the CHARMM all-atom
additive biological force fields. J. Comput.
Chem..

[42] Jorgensen W. L., Chandrasekhar J., Madura J. D., Impey R. W., Klein M. L. (1983). Comparison
of Simple Potential Functions for Simulating Liquid Water. J. Chem. Phys..

[43] Darden T., York D., Pedersen L. (1993). Particle mesh Ewald: An N·log­(N)
method for Ewald sums in large systems. J. Chem.
Phys..

[44] Essmann U., Perera L., Berkowitz M. L., Darden T., Lee H., Pedersen L. G. (1995). A smooth particle mesh Ewald method. J. Chem. Phys..

[45] Hess B., Bekker H., Berendsen H. J. C., Fraaije J. G. E. M. (1997). LINCS: A linear
constraint solver for molecular simulations. J. Comput. Chem..

[46] Miyamoto S., Kollman P. A. (1992). Settle: An analytical
version of the SHAKE and RATTLE
algorithm for rigid water models. J. Comput.
Chem..

[47] Bussi G., Donadio D., Parrinello M. (2007). Canonical sampling through velocity
rescaling. J. Chem. Phys..

[48] Bernetti M., Bussi G. (2020). Pressure control using stochastic cell rescaling. J. Chem. Phys..

[49] Gowers, R. J. ; Linke, M. ; Barnoud, J. ; Reddy, T. ; Melo, M. N. ; Seyler, S. L. ; Domanski, J. J. ; Dotson, D. L. ; Buchoux, S. ; Kenney, I. M. ; Beckstein, O. MDAnalysis: A Python Package for the Rapid Analysis of Molecular Dynamics Simulations In SciPy 2016.

[50] McGibbon R. T., Beauchamp K. A., Harrigan M. P., Klein C., Swails J. M., Hernández C. X., Schwantes C. R., Wang L. P., Lane T. J., Pande V. S. (2015). MDTraj: A Modern Open Library for the Analysis of Molecular
Dynamics Trajectories. Biophys. J..

[51] Smart O. S., Neduvelil J. G., Wang X., Wallace B. A., Sansom M. S. (1996). HOLE: a
program for the analysis of the pore dimensions of ion channel structural
models. J. Mol. Graph.

[52] Eberhardt J., Santos-Martins D., Tillack A. F., Forli S. (2021). AutoDock Vina 1.2.0:
New Docking Methods, Expanded Force Field, and Python Bindings. J. Chem. Inf. Model..

[53] Bolton E. E., Chen J., Kim S., Han L., He S., Shi W., Simonyan V., Sun Y., Thiessen P. A., Wang J., Yu B., Zhang J., Bryant S. H. (2011). PubChem3D: a new resource for scientists. J. Cheminf..

[54] Halgren T. A. (1996). Merck molecular
force field. I. Basis, form, scope, parameterization, and performance
of MMFF94. J. Comput. Chem..

[55] Perini D. A., Aguilella-Arzo M., Alcaraz A., Perálvarez-Marín A., Queralt-Martín M. (2022). Dynorphin A induces membrane permeabilization
by formation of proteolipidic pores. Insights from electrophysiology
and computational simulations. Comput. Struct.
Biotechnol. J..

[56] Guo H. C., Sun S. Q., Sun D. H., Wei Y. Q., Xu J., Huang M., Liu X. T., Liu Z. X., Luo J. X., Yin H., Liu D. X. (2013). Viroporin activity and membrane topology of classic
swine fever virus p7 protein. Int. J. Biochem.
Cell Biol..

[57] Largo E., Gladue D. P., Torralba J., Aguilella V. M., Alcaraz A., Borca M. V., Nieva J. L. (2018). Mutation-induced
changes of transmembrane pore size revealed by combined ion-channel
conductance and single vesicle permeabilization analyses. Biochim. Biophys. Acta, Biomembr..

[58] Smart O. S., Goodfellow J. M., Wallace B. A. (1993). The pore dimensions of gramicidin
A. Biophys. J..

[59] Walters R. F. S., DeGrado W. F. (2006). Helix-packing motifs
in membrane proteins. Proc. Natl. Acad. Sci.
U. S. A..

[60] Adamian L., Liang J. (2001). Helix-helix packing and interfacial pairwise interactions of residues
in membrane proteins. J. Mol. Biol..

[61] Karlin A., Akabas M. H. (1998). Substituted-cysteine accessibility method. Methods Enzymol..

[62] Dowhan W., Bogdanov M. (2012). Molecular genetic and
biochemical approaches for defining
lipid-dependent membrane protein folding. Biochim.
Biophys. Acta, Biomembr..

[63] Piersimoni L., Kastritis P. L., Arlt C., Sinz A. (2022). Cross-Linking Mass
Spectrometry for Investigating Protein Conformations and Protein–Protein
Interactions—A Method for All Seasons. Chem. Rev..

