# Supplementary material for: Structural Bases for the Unconventional Activity of a Viroporin Channel
Source: Biochemistry. 2026 Jun 25;65(13):2108–22. doi: 10.1021/acs.biochem.6c00159 (PMC13348033; doi:10.1021/acs.biochem.6c00159)
Supplement: Supplementary file 1 [file bi6c00159_si_001.pdf]

Supporting Information

Structural Bases for the Unconventional Activity of a Viroporin Channel

Brian Wiley[1], Eneko Largo[2], Laura Nabais[1], José L. Nieva[3,4*] and Carmen Domene[1*]

[1]Department of Chemistry, University of Bath, Claverton Down, Bath, BA2 7AX, United Kingdom.
[2]Department of Immunology, Microbiology and Parasitology, Medicine and Odontology Faculty, University of Basque Country (EHU), PO Box 644, 48080 Bilbao, Spain.
[3]Instituto Biofisika (CSIC, EHU), University of the Basque Country (EHU), P.O. Box 644, 48080 Bilbao, Spain
[4]Department of Biochemistry and Molecular Biology, University of the Basque Country (EHU), P.O. Box 644, 48080 Bilbao, Spain
*Correspondence to: JLN (email: joseluis.nieva@ehu.eus) and CD (email: C.Domene@bath.ac.uk)

Table S1. Summary of CSFV p7 oligomeric models generated in this study. Models correspond to TMH2-based assemblies derived from ColabFold predictions, including pentameric (high-confidence), hexameric, and heptameric configurations, as well as a low-confidence control model. Reported quality metrics include mean pLDDT, predicted aligned error (PAE), pTM, and ipTM scores. The pLDDT score reflects confidence in the local structure of individual residues, whereas PAE estimates uncertainty in the relative positioning of residue pairs and subunits. The pTM score provides a global estimate of overall fold accuracy, while the ipTM score evaluates confidence in inter-subunit interfaces and is therefore particularly informative for assessing the plausibility of the predicted oligomeric assemblies.

Model	Name	Generation	pLDDT	PAE[a]	pTM	ipTM
**$TMH2_5$**	$TMH2_5$ / high_pLDDT	ColabFold v1.6.1	75	13.3	0.55	0.49
**$TMH2_6$**	$TMH2_6$ / high_pLDDT	ColabFold v1.5.5	83	10	0.69	0.65
**$TMH2_7$**	$TMH2_7$ / high_pLDDT	ColabFold v1.5.5	80	13	0.60	0.55
**$TMH2_6L$**	$TMH2_6L$ / low_pLDDT	ColabFold v1.6.1	52	22	0.26	0.15

[a]Corresponding PAE plots are displayed below

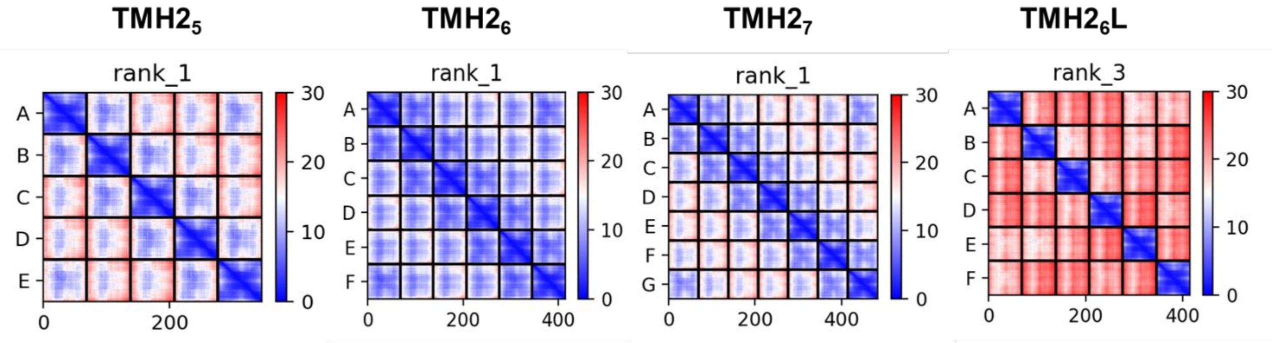

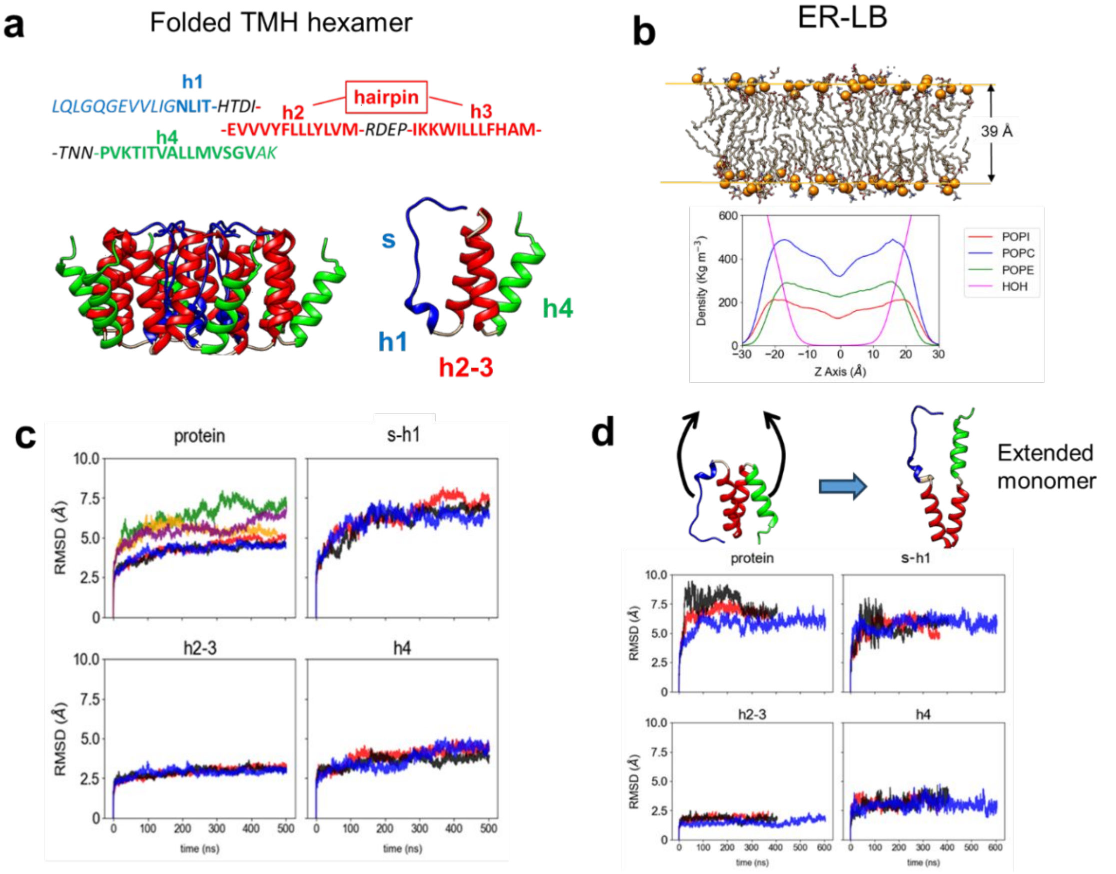

Figure S1. Systems used to build models for MD simulations **(a)** Sequence of CSFV p7 (top) and AlphaFold structure of the hexamer and composing protomers used as templates in this study (bottom) **(b)** Atomistic model of the endoplasmic reticulum lipid bilayer (ER-LB). Structure and density of the lipid components are displayed on top and bottom panels, respectively. **(c)** RMSD changes over time in the membrane-inserted protein and its different components, *i.e.* s-h1, the h2–3 hairpin, and h4, as indicated in the panels. Three replicas are shown, each depicted in a different colour (blue, red, and black traces). The full-protein panel also includes traces monitoring RMSD changes in solution (green, orange, and magenta). **(d)** Generation (top) and stability in ER-LB membranes (bottom) of the extended hairpin monomers used to build the TMH1 model (see main text).

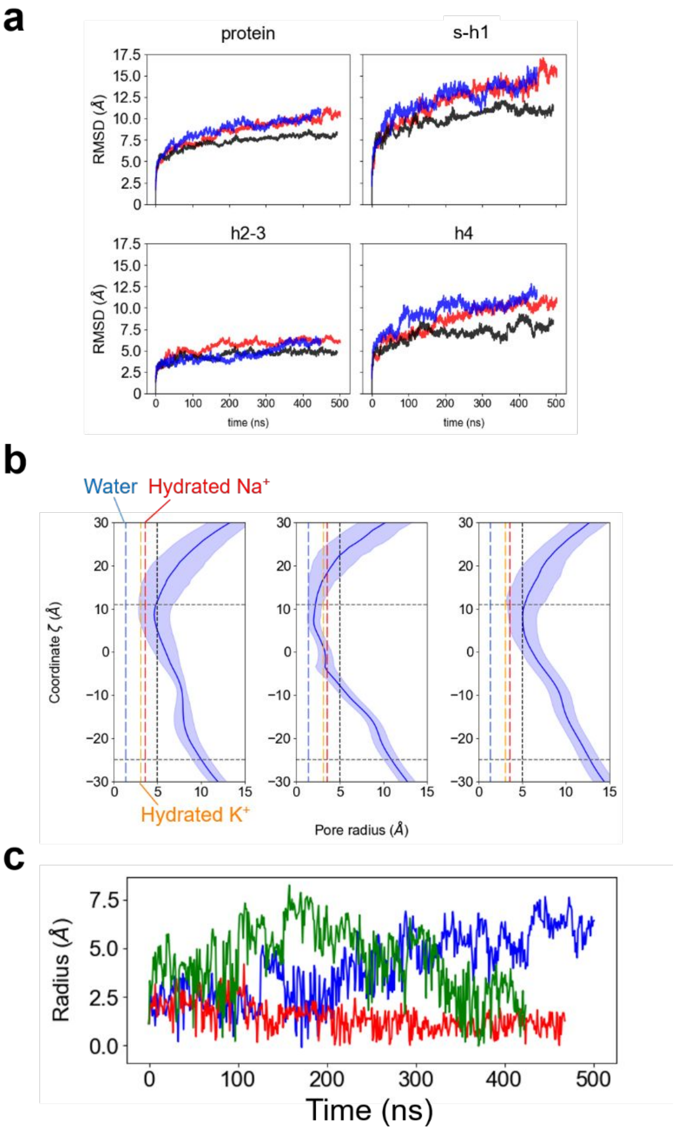

Figure S2. Stability and pore-forming activity of the TMH1 hexamer model in the ER-LB system with KCl. (a) RMSD changes during the simulations. **(b)** HOLE-calculated, time-averaged pore radius profiles in three different simulations. Blue, red and orange dashed lines indicate radii of water and hydrated Na^+ and K^+ respectively. The black dotted line marks a 1-nm-diameter pore. **(c)** Evolution of the pore radius over the simulation time. Three different replicas are shown in different colours.

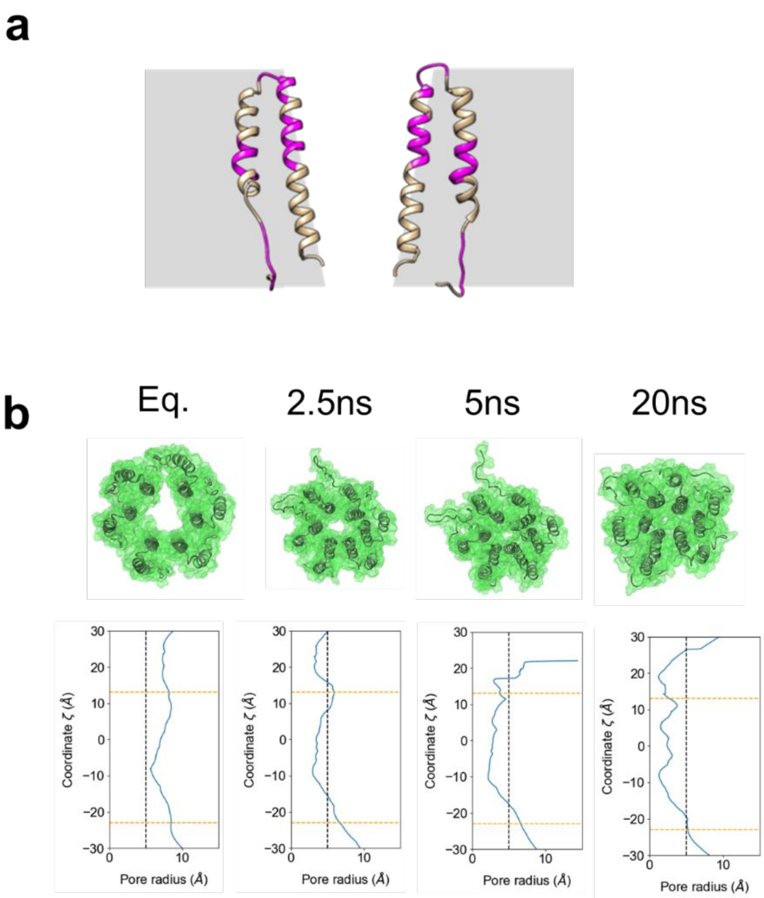

Figure S3. MD simulations on a previously proposed CSFV p7 model. (a) Simplified two-helix structure based on the model proposed by Gladue et al.[1] (b) Fast collapse of the initial pore during the simulation in ER-LBs. Shown snapshots and HOLE pore profiles correspond to the indicated equilibration and simulation times. Black dashed lines indicate a diameter of 1 nm. Orange dashed lines indicate the membrane boundaries.

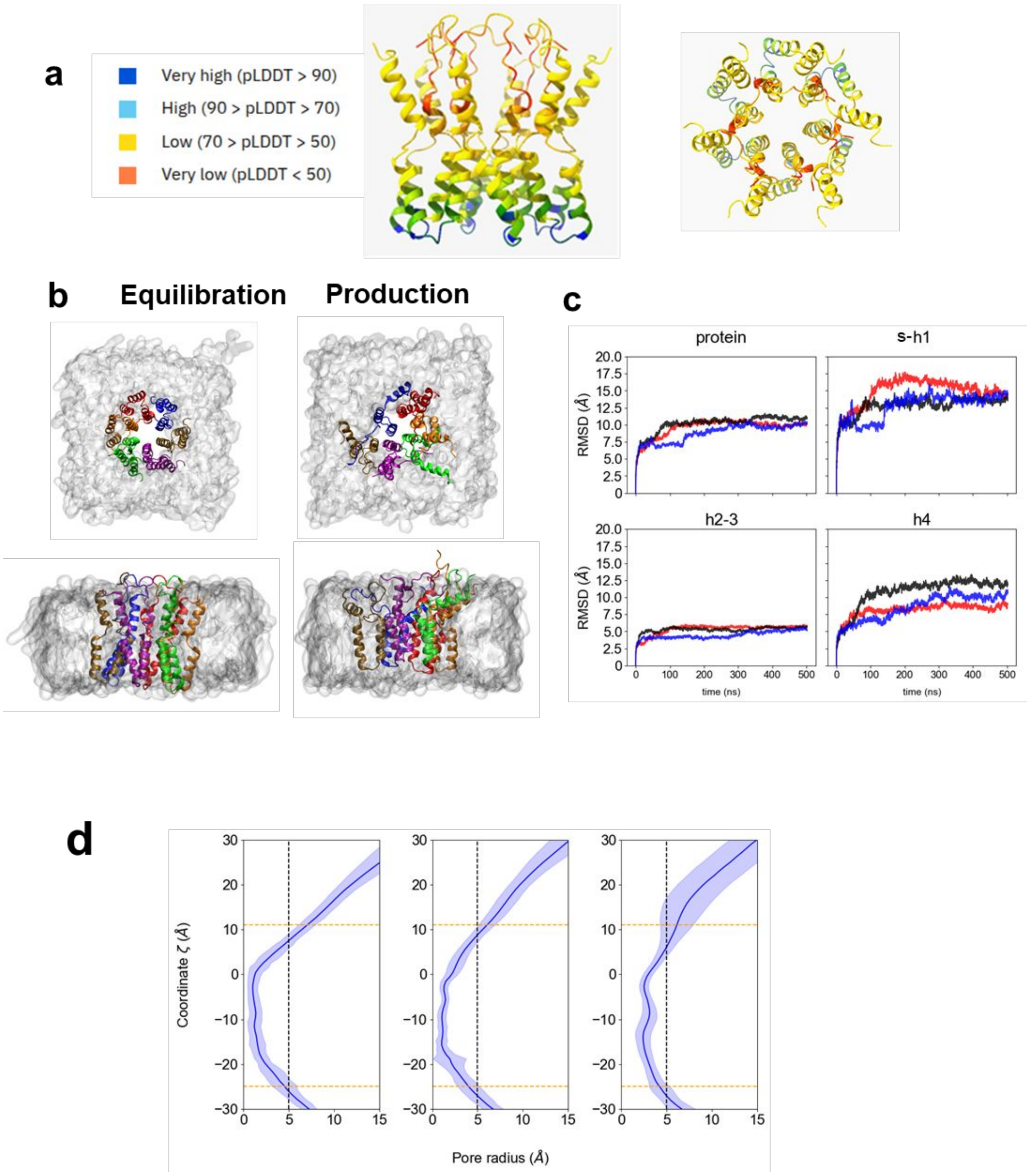

Figure S4. Stability and pore-forming activity in the ER-LB system of the extended TMH2$_6$L hexamer model rendered with low pLDDT score. (a) Structural model from ColabFold v1.5.5. **(b)** Top and side-views of the model inserted into the ER-LB membrane with each monomer colored differently. **(c)** Evolution of the protein RMSD values with simulation time for each of the three replicas shown in different colors. **(d)** Time-averaged pore-radius profiles calculated using HOLE for three replicas. Black dashed lines indicate a radius of 5 Å. Orange dashed lines indicate the membrane boundaries.

References

[1] Gladue, D. P.; Holinka, L. G.; Largo, E.; Fernandez Sainz, I.; Carrillo, C.; O'Donnell, V.; Baker-Branstetter, R.; Lu, Z.; Ambroggio, X.; Risatti, G. R.; et al. Classical swine fever virus p7 protein is a viroporin involved in virulence in swine. *J Virol* **2012**, *86* (12), 6778-6791.
